# The role of *HIL1* in strain-level adhesion and immune recognition in *Debaryomyces hansenii*

**DOI:** 10.1128/mbio.01107-26

**Published:** 2026-07-30

**Authors:** Kevin P. Newhall, Ana V. de Sousa Bezerra, Jay M. Goddard, Sarah K. McNeer, Aditi Mittal, Daniel J. Gutzmann, Supraja Swamy, Meenal Karuvannur, Scott T. Espenschied, Julie Y. Zhou, Kurt W. Runge, Jacob A. Kurowski, Deborah A. Hogan, Thaddeus S. Stappenbeck

**Affiliations:** 1 Department of Inflammation & Immunity, Cleveland Clinic Research, Cleveland, Ohio, USA; 2 Department of Pathology, Case Western Reserve University School of Medicine 12304, Cleveland, Ohio, USA; 3 Department of Microbiology and Immunology, Geisel School of Medicine at Dartmouth, Hanover, New Hampshire, USA; 4 Division of Pediatric Gastroenterology, Hepatology & Nutrition, Cleveland Clinic Children’s, Cleveland, Ohio, USA; 5 Cleveland Clinic Lerner College of Medicine, Case Western Reserve University 2546, Cleveland, Ohio, USA; Instituto Carlos Chagas, Curitiba, Brazil

**Keywords:** *Debaryomyces hansenii*, adhesin, biofilm, strain diversity, genetic screen, hydrophobicity, serum, Crohn disease

## Abstract

Strains of food-derived microbes can become facultative pathogens in susceptible human hosts. Surprisingly, we previously isolated *Debaryomyces hansenii*, a yeast common in fermented foods, from Crohn disease (CD) ulcers, raising questions about its strain-specific traits that influence host interactions. Here, we further developed the genetic tractability of *D. hansenii* and identified a single adhesin, Hil1, as a major determinant of colony morphology, biofilm formation, and immune targeting in CD patients. We used *Agrobacterium tumefaciens*-mediated transformation to perform a forward genetic screen in a food-derived reference strain. We isolated mutants that converted from a wrinkled, biofilm-forming phenotype to a smooth, non-adherent phenotype characteristic of CD patient isolates. Mapping of multiple insertion sites showed a disrupted subtelomeric Hyr/Iff-like adhesin gene, herein referred to as *HIL1*. CRISPR-Cas9-mediated deletion of *HIL1* recapitulated the mutant phenotype, demonstrating that *HIL1* was necessary for biofilm formation and high cell-surface hydrophobicity phenotypes. To contextualize these findings, we performed comparative genomics on a *D. hansenii* strain collection to assess allelic variation in the number of *HIL1* tandem repeats. Longer alleles in food strains correlated with increased biofilm formation, while CD-isolated strains contained shorter *HIL1* alleles and reduced binding to surfaces. Serology profiling showed that *HIL1* was a direct antigenic target of circulating immunoglobulin G (IgG) in CD patients. Together, these results suggest Hil1 is a key, strain-variable adhesin shaping fungal surface properties and host immune recognition. This work establishes *D. hansenii* as a genetically tractable system and shows how adhesin polymorphisms may influence fungal behavior in food and disease contexts.

**IMPORTANCE:**

*Debaryomyces hansenii* is a yeast that is common in food and is generally recognized as safe for human consumption, though recently it has been identified within diseased regions of the intestine in Crohn disease patients. A current need is to determine the genetic and phenotypic differences between safe food isolates and isolates from human Crohn disease patient ulcers. Here, we used a loss-of-function genetic screen and identified *HIL1*, an adhesin that we found mediates cellular adhesion in many food strains but not in patient strains. We identified circulating *HIL1*-reactive antibodies in patients with Crohn disease, indicating that food strains can be a target of host immune responses through Hil1.

## INTRODUCTION

Many of the foods we eat are colonized by live microbes, such as yeasts, which in turn affect the species detected in the fecal microbiome (1). Whether these food microbes simply pass through the gastrointestinal tract or impact the host is not well understood. *Debaryomyces hansenii* is a yeast found in many kinds of cheese (2) and is commonly detected in the human gut mycobiome through fecal sequencing and culture-based methods in both healthy and diseased subjects (1, 3–10). While *D. hansenii* is considered generally recognized as safe as a food additive, our group recently identified culturable *D. hansenii* within intestinal ulcerations of patients with Crohn disease (CD) (10). These *D. hansenii* CD isolates inhibited epithelial repair in a mouse model of intestinal injury, similar to non-healed wounds in CD, while another common food microbe, *Saccharomyces cerevisiae,* did not. These experiments fulfilled key aspects of Koch’s postulates, supporting a role for *D. hansenii* in CD. Genetic and phenotypic diversity between strains of the same food fungi can be extensive (11). *D. hansenii* is similar in this respect, with food strains exhibiting genetic variation in total chromosome number and changes to ploidy, resulting in wide phenotypic diversity (12–16). Key unanswered questions include (i) whether phenotypic differences exist comparing generally recognized as safe food strains and CD patient isolates of *D. hansenii*, (ii) whether these phenotypes can be interconverted experimentally, and (iii) the nature of the underlying genetic basis of these differences.

A known source of strain diversity in fungi is adhesins, which are a class of cell wall proteins that facilitate the formation of microbial biofilms or attachment to substrates, such as extracellular matrix or host epithelial cells (17, 18). An enrichment in the number of adhesin genes appears to occur in pathogenic yeast species as compared to less pathogenic ones (17, 19–22). As cell wall proteins, adhesins are common antigens for antibody targeting (23). Expression of large repertoires of adhesins can therefore be a tradeoff for fitness within a host, as antibody responses targeting adhesins can lead to neutralization and clearance (23–25). *In silico* analyses of fungal adhesins in the *D. hansenii* reference genome CBS767 identified three ORFs predicted to encode adhesin proteins, all belonging to the Hyr/Iff-like (HIL) adhesin family (19, 21). While observational studies of *D. hansenii* food strains have shown heterogeneity of cellular adhesion (26, 27), the identity of the gene or genes that govern these phenotypes has not been determined experimentally.

Additional methods that permit more facile mutational analyses toward unbiased genetic screening in *D. hansenii* are needed. Genetic manipulations in *D. hansenii* are challenging (28), but recently Strucko et al. generated single gene knockouts using CRISPR-Cas9 editing in a non-homologous end joining deficient mutant of *D. hansenii* (29). We and others generated deletion mutants in non-model fungi through a synthetic Cas9 ribonucleoprotein (RNP) delivery system, though this method has not yet been adapted to *D. hansenii* (30, 31). *Agrobacterium tumefaciens*-mediated transformation (AtMT) (32) is an insertional mutagenesis platform that can identify genes governing fungal pathogenicity, such as melanin or capsule, in *Cryptococcus neoformans* (33, 34), as well as adhesion molecules in *Candida auris* (35). These screens leverage simple, observational phenotypes to efficiently identify genetic determinants (36). Paired with reverse genetic validation of screen hits, these systems can facilitate the tractability of microbes of emerging significance. Genetic tractability is particularly important in this age of microbiome sequencing, which often identifies non-model organisms associated with complex host phenotypes, but lacks the tools needed to functionally validate their role in disease (37).

The coupling of genetic manipulation in model strains with phenotypic analysis of strain collections shows microbial mechanisms that are relevant to human diseases like inflammatory bowel disease (IBD). In the gut pathobiont *Candida albicans*, screening a cohort of genetic mutants from the laboratory strain SC5314 for the capacity to induce epithelial cell death led to the discovery of the toxin candidalysin (38). This laid the foundation for subsequent groups to study whether candidalysin, which is linked to the hyphal form of *C. albicans*, is altered in the gut during health and disease states. Through screening a collection of human IBD-derived isolates of *C. albicans*, Li et al. showed strain variation in candidalysin expression between isolates dictated colitogenic Th17 responses (39). Taken together, these findings highlight the necessity of genetic tractability and strain diversity analysis in furthering our understanding of the microbial factors that influence disease.

Here, we define a strategy to ascertain the phenotypic diversity of *D. hansenii* strains in the context of CD-correlated phenotypes. We used colony morphology phenotypes to identify surface factors that impact host interactions. We utilized forward genetic screening and reverse genetic validation to uncover a role for an adhesin that we named *HIL1* in governing colony morphology and biofilm formation in *D. hansenii*. We created and sequenced a *D. hansenii* strain collection comprising nine food and three CD-patient isolates and used comparative genomics to identify polymorphisms in *HIL1* that affect protein structure and function. Finally, we showed that human CD patients develop serum antibodies that bind *D. hansenii* strains, and that Hil1 was a target. Thus, our efforts to further develop the genetic tractability of *D. hansenii* yielded new insights into the differential abundance of cell wall proteins that govern differential biofilm formation among food and patient strains of *D. hansenii*. Ultimately, this work identified microbial factors associated with food that are targeted by the human adaptive immune system in the setting of CD.

## RESULTS

### Microbial phenotypic variation between food- and patient-derived *D. hansenii* isolates

**Fig 1:**
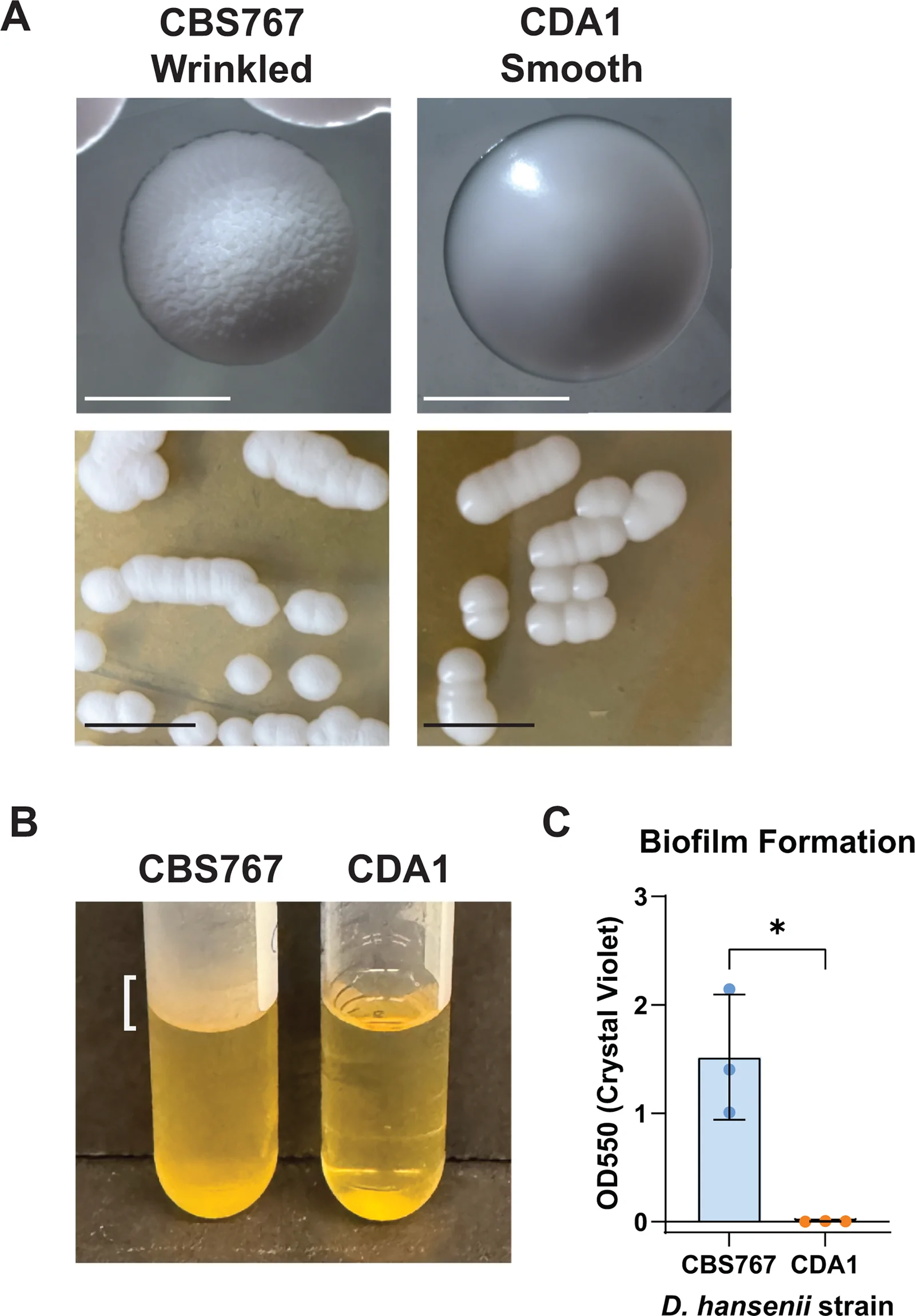
Phenotypic differences between a representative food strain (CBS767) and a representative patient strain (CDA1). (**A**) Representative photographs of colony morphology for each indicated strain. The upper panels show single colonies to display the wrinkled and smooth morphologies. The lower panels are lower-power views of multiple colonies to show consistent morphology. Bars = 1 mm (white, upper panels) and 10 mm (black, lower panels). (**B**) Representative photograph of saturated cultures of indicated strains grown in YPD medium and remained in culture statically for one additional day. The white bracket highlights floating cells at the air-liquid interface in the CBS767 culture, consistent with pellicle formation. (**C**) Quantification of biofilm formation of the indicated strains. *Y*-axis is a plot of absorbance spectroscopy at OD550 of solubilized crystal violet treatment of a 24 h culture on polystyrene plates. * denotes *P* < 0.05. Error bars, SD. Significance was determined by a paired Student’s *t*-test, t(2) = 4.543, *P* = 0.0452 from three independent experiments.

Analysis of phenotypic diversity within a species can aid in the identification of factors important for host interactions. We assessed whether CD-derived and food-derived isolates of *D. hansenii* differed phenotypically. We first selected two prototypic strains for comparative phenotypic analysis: the genomic reference strain CBS767, originally cultured from a food source, and the CD patient intestinal ulcer strain CDA1 (10). When grown on yeast peptone dextrose (YPD) agar, we observed differences in the predominant colony morphology between these two strains: CBS767 displayed a wrinkled (Wr) colony morphology, defined by furrows on the colony surface that resulted in a dull, matte appearance, while CDA1 displayed a smooth (Sm) colony morphology, defined by a uniform appearance of the colony surface that was shiny and reflected light (Fig. 1A). Growth of each strain in liquid YPD medium overnight to dense cultures, followed by an additional 24 h of undisturbed aerobic growth, showed that CBS767 formed a visible pellicle at the air-liquid interface of the test tube, while CDA1 did not (Fig. 1B). Pellicle formation is a biofilm phenotype often selected for by long-term fermentative cultures, such as sherry, from which CBS767 was originally derived (40). Notably, the relationship between the Wr colony morphology and pellicle formation is conserved in other food-relevant yeasts like *S. cerevisiae*; genetic deletion of cell wall proteins, such as the *FLO11* adhesin, can convert colonies from Wr to Sm (41, 42). Pellicle formation factors also often impact the formation of biofilms on abiotic surfaces (43). Thus, we also measured biofilm formation in these two strains by a crystal violet assay, staining 96-well polystyrene plates after 24 h of undisturbed culture (44). We observed the formation of biofilms by strain CBS767 but not patient strain CDA1 (Fig. 1C). This was not associated with differences in growth rate between the two strains (Fig. S1A). These data suggest a loss of a food-relevant phenotype, namely pellicle biofilm formation, in the patient-derived strain of *D. hansenii,* which appears to correlate with colony morphology.

### Forward genetic screening identifies mutants that convert Wr to Sm in morphology

**Fig 2:**
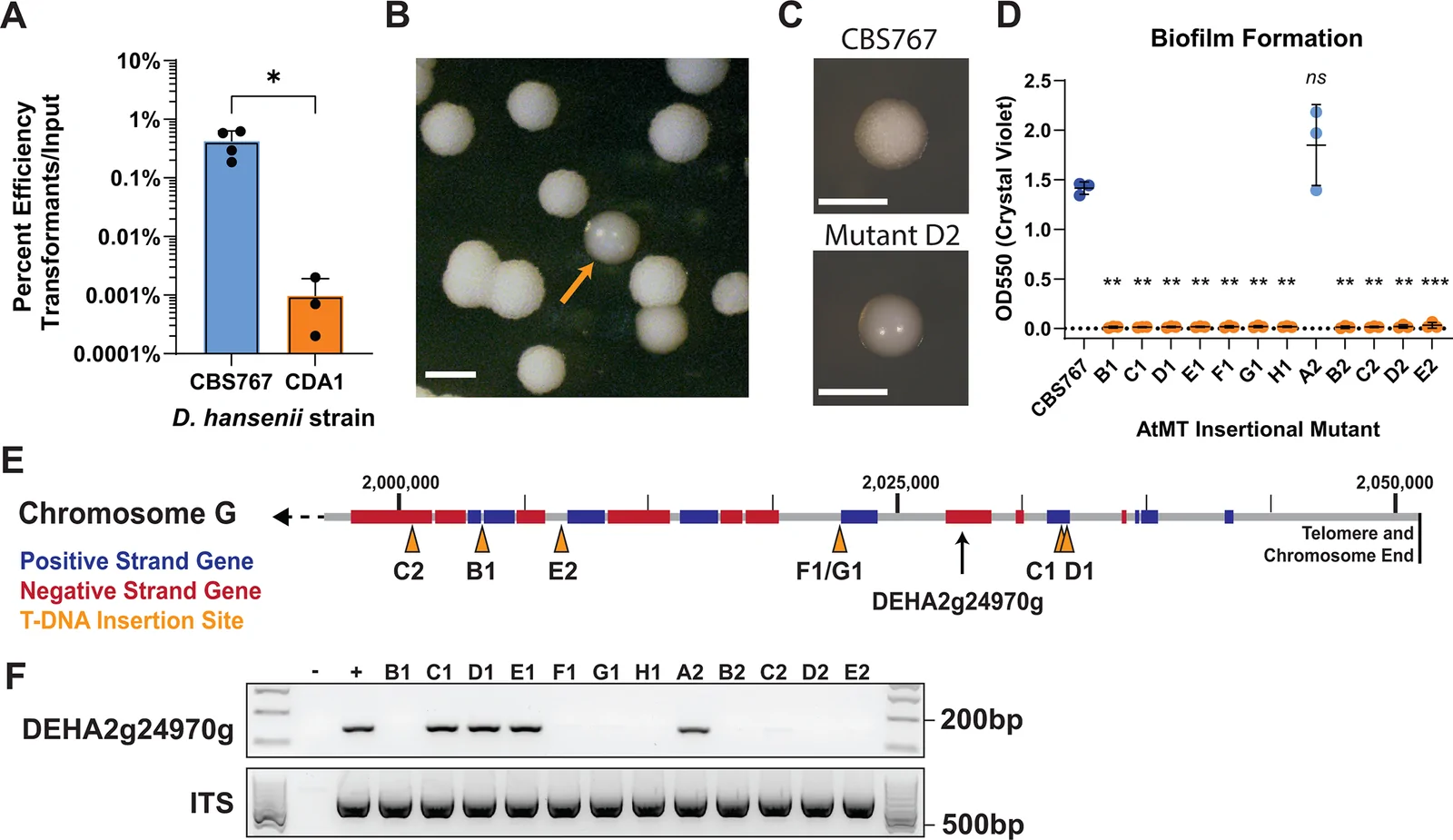
*A. tumefaciens-*mediated transformation, insertional mutagenesis, and forward genetic screen identify CBS767 transformants that convert from wrinkled to smooth phenotype. (**A**) Quantification of percent efficiency (transformants/total input) of nourseothricin-resistant colonies recovered following AtMT for the indicated *D. hansenii* strains. Each dot represents an experimental replicate. CBS767 had an average of 382 colonies per plate per transformation, while CDA1 had an average of one colony per plate per transformation. (**B**) Representative photograph of colony morphology mutants selected via forward genetic screen. The orange arrow highlights a mutant on nourseothricin selection agar with smooth morphology. Bar = 1 mm. (**C**) Representative photographs of mutant D2 following glycerol stock revival compared to parent strain (CBS767). Bar = 1 mm. (**D**) Quantification of biofilm formation of insertional mutants by crystal violet assay (polystyrene adhesion assay). Insertional mutant A2 served as Wr negative control; remaining mutants exhibited Sm morphology. Each point denotes an independent biological replicate. (**E**) Schematic map of t-DNA insertions to the terminal region of chromosome G of *D. hansenii* CBS767 reference genome, adapted from FungiDB. Orange triangles denote the insertion site of t-DNA, blue squares denote positive-strand genes, and red squares denote negative-strand genes. Black lines indicate chromosomal position in base pairs. (**F**) SYBR safe-stained agarose gel of indicated PCR products. Genomic DNA was used as a template for PCR amplification of a 136 bp amplicon within the coding sequence of DEHAg24970g. As a loading control, the ITS1-5.8SrRNA-ITS2 (ITS) region was amplified. The negative control (−) is no DNA (water), the positive control (+) is parental CBS767 genomic DNA. Statistical analysis. * denotes *P* < 0.05, ** denotes *P* < 0.01, *** denotes *P* < 0.001, and “ns” denotes *P* > 0.05. Error bars, SD. (**A**) Significance was determined by Welch’s *t*-test, t(3) = 3.914, *P* = 0.0296. (**D**) Significance determined by Welch’s ANOVA test, W(12.00, 10.03) = 74.80, *P* < 0.0001 with Dunnett’s T3 multiple comparisons test plotted.

Given the clear differences in colony morphology between CBS767 and CDA1, we sought to perform an unbiased conversion of a Wr strain (CBS767) to a Sm phenotype, characteristic of patient strains. To accomplish this goal, we utilized the AtMT system, adapted from *C. auris* (45), to produce insertional mutants in a Wr *D. hansenii* strain. Importantly, we observed efficient transformation of the Wr strain CBS767 (0.4%; Fig. 2A), enabling a forward genetic screen to identify mutants that transitioned from a parental Wr phenotype to a Sm phenotype.

Following AtMT in the Wr strain CBS767, we screened more than 5,000 nourseothricin-resistant colonies using a stereomicroscope to assess morphology. Seventeen Sm mutants were identified (Fig. 2B), corresponding to a conversion rate of approximately 0.3%. This phenotype remained stable after revival from glycerol stock storage (Fig. 2C). For downstream analysis, we selected 12 representative mutants: 11 were Sm, and 1 remained Wr as a negative control. Remarkably, all 11 Sm insertional mutants (B1-E2) failed to form biofilms on polystyrene, while the negative control Wr insertional mutant A2 retained this capacity (Fig. 2D). This was not associated with changes in growth rate (Fig. S1B). These results support a link between *D. hansenii* Wr colony morphology and biofilm formation on polystyrene.

To identify the candidate mutations associated with Sm colony morphology and loss of biofilm formation, we mapped t-DNA insertions in the mutants tested in Fig. 2D using pooled whole-genome sequencing as previously described (45). Each strain per pool was genotyped via PCR spanning the insertion site to deconvolute pooled mutants (Table S1). Notably, six unique transposon mutants were mapped to the subtelomeric region (~50 kb) of *D. hansenii* ChrG (Fig. 2E).

To better understand the nature of the enrichment of mutants in the subtelomeric region of ChrG in our t-DNA hits, we evaluated flanking regions to the mutant. While mapping t-DNA flanking contigs to the genome, we observed cases where the left t-DNA border would map to a locus on ChrG, but no matching sequence could be identified on the right t-DNA border; instead, tandem repeat sequences, consistent with telomeres, were identified. This suggested that t-DNA insertion could result in total distal deletions to the chromosome tip (Fig. S2). To evaluate this possibility, we performed PCR amplification of DEHA2g24970g, one of the most distal genes on ChrG. We failed to observe amplification of a DEHA2g24970g product in 8/11 Sm mutants from our screen, consistent with deletion of this region of the chromosome, and supporting that t-DNA insertion could result in complex deletions of multiple genes (Fig. 2F).

### Identification of *D. hansenii* adhesin *HIL1*

To identify which of the 20 genes in the subtelomeric locus accounted for the loss of biofilm production on polystyrene, we prioritized genes that encoded for cell wall proteins as the most likely mediators of adhesion phenotypes. Using FungiDB (46), we filtered the *D. hansenii* CBS767 reference genome for genes containing predicted signal peptides, which identified 295 candidate genes. Two predicted genes were located within the subtelomeric region of chromosome G: DEHA2G24970g and DEHA2G25124g. Additional analysis of DEHA2G25124g showed it lacked a predicted GPI anchor (NetGPI-1.1, likelihood score of 0.988 being not GPI) (47), suggesting it was secreted rather than cell wall anchored. In contrast, DEHA2G24970g was predicted to contain a GPI anchor, as well as the Hyphal_Reg_CWP protein domain, an adhesin motif present within the Hyr/Iff families of cell wall proteins in *C. albicans*. Based on this analysis, DEHA2G24970g emerged as the top candidate gene mediating the differences in colony morphology and pellicle formation in the mutant strains of *D. hansenii*.

Comparative genomic analyses identified three ORFs containing predicted Hyphal_Reg_CWP protein domains in the CBS767 reference genome, including DEHA2G24970g, DEHA2B01452g, and DEHA2D00132g (19, 21). Of note, DEHA2G24970g was located within the subtelomeric region of ChrG, and was deleted in many of the Sm AtMT insertional mutants (Fig. 2F). Using endpoint reverse transcription (RT)-PCR, we detected mRNA expression of all three ORFs in CBS767. In contrast, CDA1 did not express DEHA2D00132g (Fig. S3A). We named these genes *HIL1* (DEHA2G24970g), *HIL2* (DEHA2B01452g), and *HIL3* (DEHA2D00132g) after the Hyr/Iff-Like (HIL) terminology used by Smoak et al., and following nomenclature rules consistent with *C. albicans* (21). Analysis of the 12 AtMT mutants for *HIL1, HIL2*, and *HIL3* expression revealed a lack of detectable *HIL1* transcripts in 8/11 phenotypic AtMT mutants (Fig. S3B), while *HIL2* and *HIL3* expression levels were similar to WT CBS767. These findings further support the conclusion that the *HIL1* adhesin underlies the alteration of colony morphology and loss of adhesion.

We also evaluated other potential genes in this locus that could affect colony morphology. The mutant E2 was a Sm convertant that carried an insertion in the promoter of DEHA2G24838g. This gene is homologous to the *BMT* family of beta-mannosyltransferases in *C. albicans*. Of note, the insertional mutant E2 also exhibited *HIL1* deletion, which is more distal than the insertion site on the chromosome (Fig. 2F). To rule out any changes to the glycan structure of the cell wall based on loss of function, cell wall extracts were analyzed via glycosyl linkage analysis and combined gas chromatography-mass spectrometry. We observed no differences in the relative abundance of glycan residues between strains (including mannose residues) (Fig. S4). These data provide additional support that Hil1 is responsible for mediating adhesion in *D. hansenii*.

### Hil1 governs adhesion through cell surface hydrophobicity

**Fig 3:**
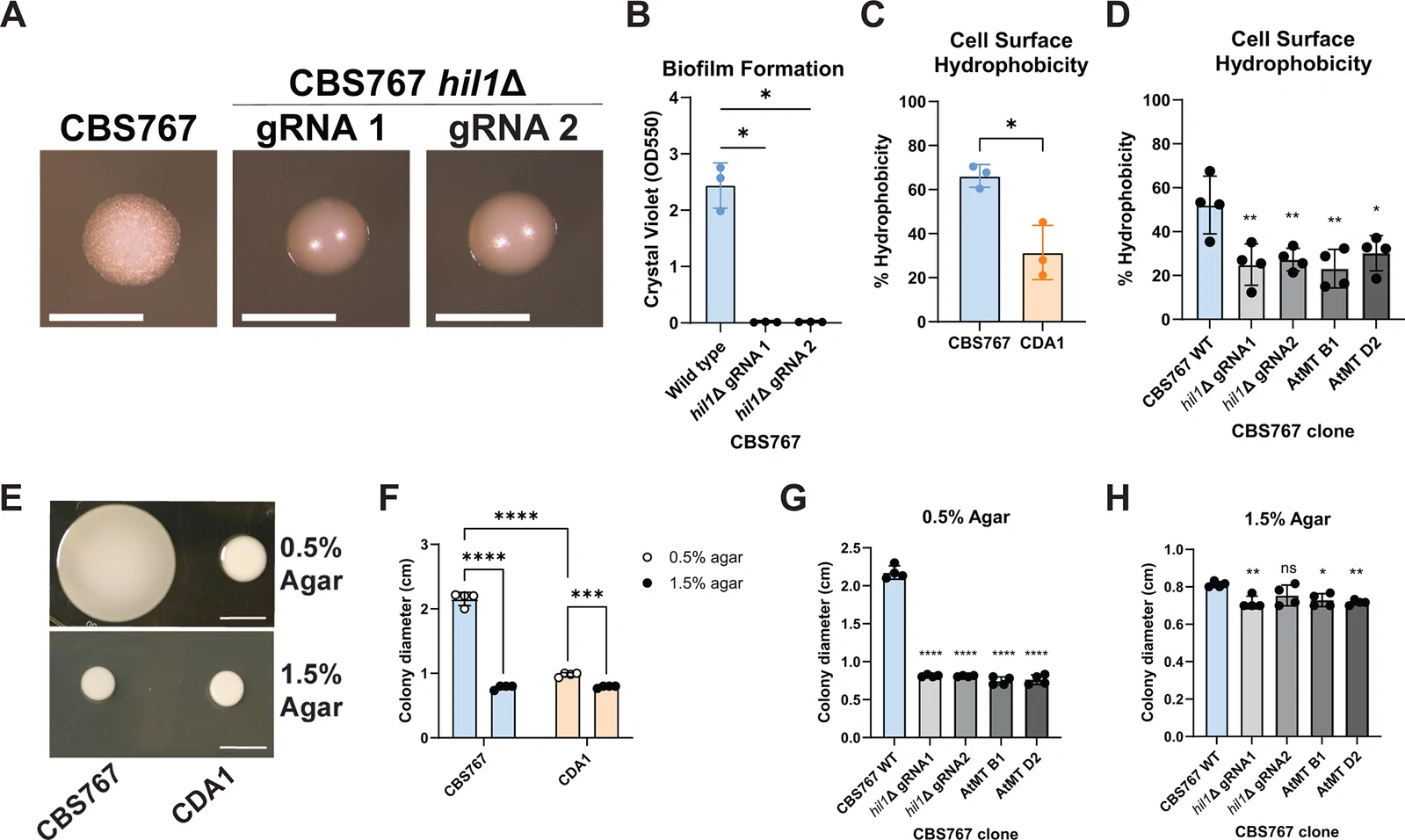
HIL1 knockout strains of CBS767 exhibit Sm colony morphology and fail to form biofilm on polystyrene or semisolid agar. (**A**) Photographs of representative colony morphologies of CBS767 *hil1*Δ strains generated with two distinct *HIL1* targeting gRNAs. Bars = 1 mm. (**B**) Quantification of 24-h biofilm formation of CBS767 *hil1*Δ mutants by crystal violet assay (polystyrene adhesion assay). (**C and D**) Cell surface hydrophobicity of indicated strains determined by microbial adhesion to hydrocarbons (MATH) assay. (**E**) Representative photograph of indicated strains grown on 0.5% agar or 1.5% agar plates for 48 h. Bars = 1 cm. (**F–H**) Quantification of colony diameter under indicated conditions and yeast genotypes. Statistical analysis: each point represents an independent biological replicate. * denotes *P* < 0.05, ** denotes *P* < 0.01, *** denotes *P* < 0.001, **** denotes *P* < 0.0001, and “ns” denotes *P* > 0.05. Error bars, SD. (**B**) Brown-Forsyth ANOVA, F(2, 2) = 109.1, *P* = 0.0021. Dunnett’s T3 multiple comparisons test. (**C**) Welch’s *t*-test, T(2.676) = 4.496, *P* = 0.0261. (**D**) One-way ANOVA between strains F(4, 15) = 6.493, *P* = 0.0031; Dunnett’s T3 multiple comparisons test. (**F**) Two-way ANOVA, strain × agar% F(1, 6) = 846.0, *P* < 0.0001. *Post hoc* testing with uncorrected Fisher’s LSD. (**G**) One-way ANOVA F(4, 15) = 517.5, *P* < 0.0001. *Post hoc* testing with Dunnett’s multiple comparisons test. (**H**) One-way ANOVA F(4, 15) = 5.633, *P* = 0.0057. *Post hoc* testing with Dunnett’s multiple comparisons test.

To directly test the hypothesis that Hil1 was required for CBS767 biofilm formation and colony morphology, we generated *HIL1* null strains of CBS767 using CRISPR-facilitated homology-directed repair. We adapted a CRISPR Cas9-Ribonucleoprotein (RNP) complex system previously utilized in *Candida lusitaniae* to introduce Cas9 and synthetic guide RNAs (gRNAs) into *D. hansenii* cells. This method utilizes Cas9-induced double-stranded breaks within the gene of interest, and transformation with a *SAT1* resistance cassette containing flanking homology arms to replace the gene of interest (30). We designed two unique gRNAs using the Alt-R RNP gene editing system (IDT) and evaluated for off-target effects to the CBS767 genome using EuPaGDT (48). Transfection of RNP complexes with the homology-directed repair (HDR) cassette resulted in nourseothricin-resistant colonies that converted from Wr to Sm morphology (Fig. 3A). PCR validation of clones isolated following transformation with either of the *HIL1*-targeting gRNAs confirmed *SAT1* replacement of *HIL1*. Relative to the parental CBS767 strain, CBS767 *hil1*Δ generated with gRNA1 or gRNA2 was unable to form biofilm (Fig. 3B). These data demonstrate that *HIL1* is required for biofilm formation in CBS767.

Cell surface hydrophobicity increases adhesion (49) and biofilm formation (50). To determine if our variable *D. hansenii* biofilm phenotypes correlated with variation in cell-surface charge, we analyzed cell surface hydrophobicity (CSH) by microbial adhesion to hydrocarbons (MATH) as previously described (51). We found that CBS767 was significantly more hydrophobic than the patient isolate CDA1 (Fig. 3C). We next tested whether CBS767 strains deficient in *HIL1*, either via CRISPR-mediated knockout or insertional mutants, displayed reduced CSH. Indeed, CBS767 *hil1*Δ strains had a roughly twofold reduction in CSH relative to wild-type CBS767 (Fig. 3D). These data suggest that *HIL1* may directly increase surface hydrophobicity, thereby promoting cell-cell and cell-surface interactions (52). We repeated our biofilm assay with CBS767 using tissue-culture treated (hydrophilic) polystyrene plates and observed a significant reduction in biofilm formation in CBS767 relative to untreated (hydrophobic) polystyrene plates (Fig. S5A). The low CSH strain CDA1 did not adhere to either plate (Fig. S5A). These findings are consistent with a role for CSH in *HIL1*-mediated adhesion in CBS767.

In addition to adhering to polystyrene, *D. hansenii* strain CBS767 can also spread on semisolid agar (27). As colony spreading is associated with the production of adhesins (53, 54), we compared CBS767 wild-type growth on semisolid agar to that of CDA1. CBS767 exhibited a roughly threefold increase in colony diameter on semisolid (0.5%) relative to solid (1.5%) agar, consistent with colony spreading (Fig. 3E and F). Strain CDA1 exhibited a minimal (1.2-fold change) increase in colony diameter on semisolid agar. Hil1 was required for this effect in CBS767, as colony diameter in *hil1*Δ CBS767 mutants was roughly threefold lower than that of the WT CBS767 strain on semisolid agar (Fig. 3G; Fig. S5B). Solid agar exhibited minimal differences in colony diameter (Fig. 3H). These data support that Hil1 can promote surface changes that allow for spreading or sliding on semisolid agar.

Cells that spread more on a semisolid surface can gain access to more nutrients, and thus, growth is promoted (55). To determine if spreading was positively correlated with increased growth, we assessed the amount of biomass in each colony. Indeed, we observed that the CBS767 wild-type strain showed significantly more growth on semisolid over solid agar compared to both CBS767 *hil1*Δ and CDA1 strains (Fig. S5C and D). Altogether, these data indicate that Hil1 functions as an adhesin in CBS767 via hydrophobic interactions. Additionally, strains CBS767 and CDA1 exhibit distinct phenotypes related to cellular adhesins, including cell surface hydrophobicity and cell-cell binding. Although loss of *HIL1* in CBS767 appears to phenocopy strain CDA1, it should be noted that CDA1 represents a distinct genetic background, which may contain additional modifiers of adhesion unrelated to *HIL1*.

### Strain allelic diversity in *HIL1*

Strain collections are essential to characterize the phenotypic diversity of a microbial species. We assembled a *D. hansenii* strain collection to better characterize phenotypic differences amongst *D. hansenii* strains, including isolates from patients with CD (10) or from the food supply (12, 56). Food strains were either obtained through isolation at the Cleveland Clinic (CCFD1-4) or acquired from previously published studies (12, 56). Regardless of the source, all strains were validated as *D. hansenii* via ITS1-5.8SrRNA-ITS2 sequencing, as well as the use of *Debaryomyces hansenii*-discriminating primers for *PAD1* (57, 58).

For each strain, we characterized colony morphology and pellicle formation (Table S2). Of note, we observed Sm colony morphology and the absence of pellicle in all three CD-derived isolates of *D. hansenii*. Food isolates exhibited a spectrum of phenotypes. Two food isolates exhibited a striking Wr morphology, four food isolates were Sm, and three food isolates exhibited an intermediate matte phenotype. Matte isolates lacked the characteristic furrows of Wr strains but did not reflect light. Importantly, the matte isolates formed pellicles at the air-liquid interface of the test tube. These data indicate that a diversity of phenotypes related to colony morphology and pellicle formation exists in food strains. Importantly, *D. hansenii* strains exist in the food supply that phenocopy the Sm morphology present in the patient-derived isolates.

**Fig 4:**
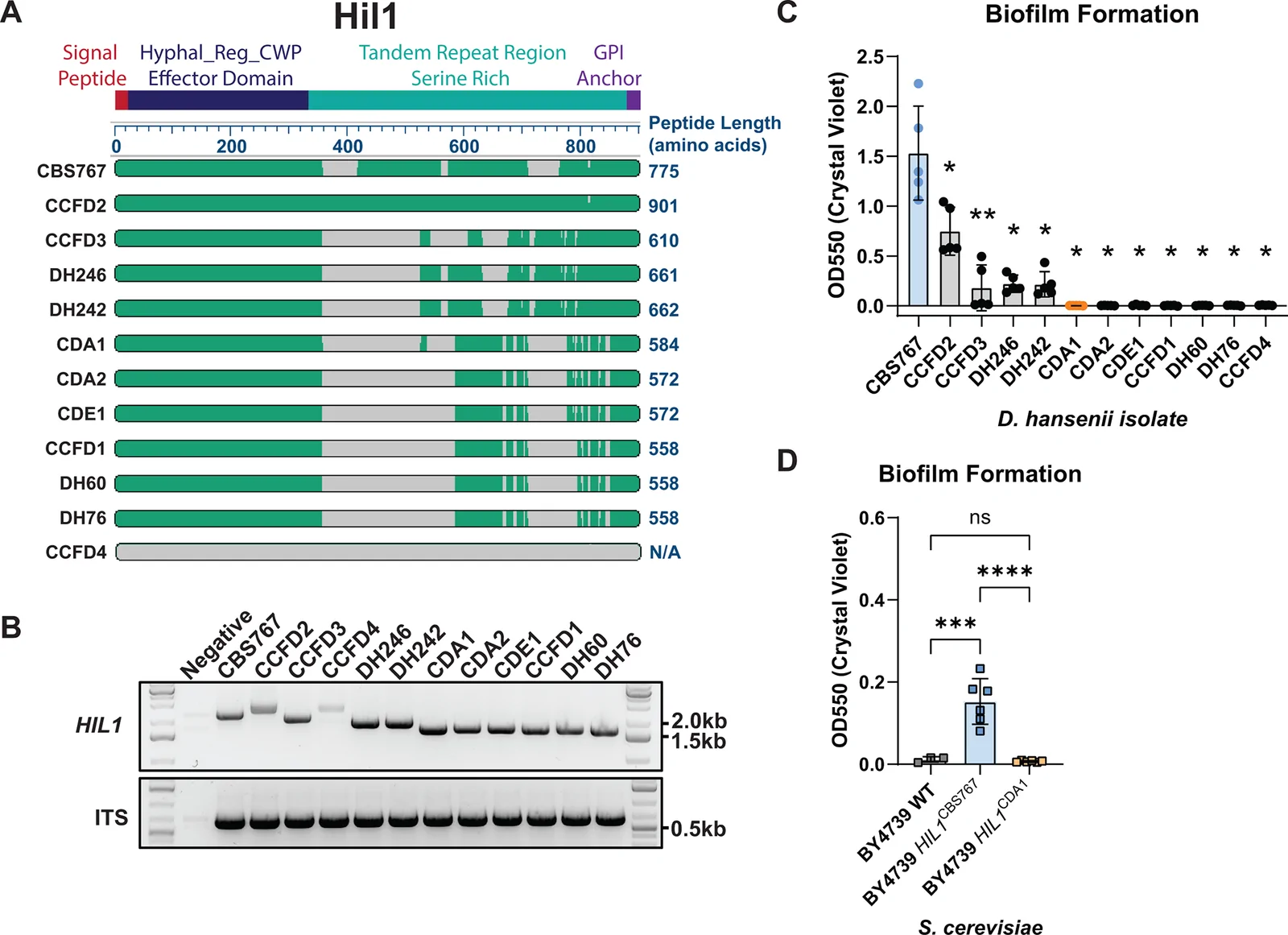
Natural variation in *D. hansenii* strain *HIL1* tandem repeat length affects hydrophobic biofilm formation. (**A**) Amino acid sequence alignment of Hil1 of the indicated strains. Green indicates sequence match; gray indicates absence of peptide. Alignment was forced to the length of CCFD2, which has additional repeats from the type strain CBS767. CCFD4 lacked an identifiable *HIL1* gene from WGS. (**B**) SYBR safe-stained agarose gel of indicated PCR products. (**C**) Quantification of 24-h biofilm formation of indicated strains by crystal violet assay (polystyrene adhesion assay). Each dot is a biological replicate. (**D**) Quantification of 24-h biofilm formation of *S. cerevisiae* with or without heterologous expression of the indicated *D. hansenii HIL1* allele. Each point is a biological replicate from two to three independent experiments. Statistical analysis: * denotes *P* < 0.05, ** denotes *P* < 0.01, *** denotes *P* < 0.001, **** denotes *P* < 0.0001, and “ns” denotes *P* > 0.05. Error bars, SD. (**C**) RM one-way ANOVA for strain F(1.084, 4.338) = 46.45, *P* = 0.0017. Holm-Šídák’s multiple comparisons test against CBS767. (**D**) One-way ANOVA F(2, 12) = 28.92, *P* < 0.0001. Tukey’s multiple comparisons test.

Whole-genome sequencing of each strain (using the CBS767 reference genome to map the reads) showed significant polymorphism within the *HIL1* gene (Fig. 4A). The total amino acid length of Hil1 varied, with a minimum of 572 amino acids, a maximum of 901 amino acids, and an interquartile range of 96.5 amino acids. Of note, the three patient isolates had consistently short, but not identical, Hil1 peptide sequences (572–584 amino acids). Importantly, *HIL1* for all strains was in frame for translation of a functional protein. Furthermore, there was high homology among the effector domain, signal peptide, and GPI anchor domains. Within the tandem repeat region, however, the number of repeats ranged widely. PCR amplification of the *HIL1* locus confirmed these size differences (Fig. 4B). Strain CCFD4 lacked a clear *HIL1* ortholog by BLAST analysis and produced a weak amplicon. The comparison of gene length to colony morphology supports the model that longer *HIL1* alleles are associated more with Wr colony morphology, and shorter alleles or the total absence of *HIL1* confer Sm morphology.

To determine whether *HIL1* allelic variation correlated with adhesion, we screened the culture collection strains of *D. hansenii* for their ability to bind polystyrene. Adhesion varied and tracked with colony morphology. CBS767 and CCFD2 (Wr strains) exhibited high binding, and all patient strains (Sm) and shiny food strains exhibited no adhesion (Fig. 4C). Matte food strains showed an intermediate level of adhesion. This pattern was similar to the pellicle formation data from each strain (Table S2). Simple linear regression of total Hil1 protein length against biofilm formation quantified via crystal violet binding revealed a moderately strong correlation, *R*^2^ = 0.6238 (Fig. S6A). These data support the hypothesis that longer alleles of Hil1 exhibit higher degrees of binding, consistent with *S. cerevisiae* adhesin *FLO1* (43).

CBS767 and CDA1 represent naturally distinct genetic backgrounds that may have confounding effects on adhesion phenotypes. To test whether allelic variation affects *HIL1*-mediated polystyrene binding, we developed a *S. cerevisiae*-based heterologous expression system to test whether the CBS767 allele (*HIL1^CBS767^*) or the CDA1 allele (*HIL1^CDA1^*) conferred adhesion in an identical genetic background. We validated that our heterologous expression system resulted in detectable *HIL1* transcript in *S. cerevisiae* cells using RT-PCR (Fig. S6B). *S. cerevisiae* strain S288C and its derivatives fail to adhere to polystyrene and have been used to test the contribution of adhesin alleles in conferring adhesion phenotypes (43). Heterologous expression of *HIL1^CBS767^* in the S288C-derived BY4739 strain of *S. cerevisiae* resulted in significantly more adhesion to polystyrene relative to WT cells or *HIL1^CDA1^*-expressing cells (Fig. 4D). Expression of *HIL1^CDA1^* did not significantly increase polystyrene adhesion relative to WT cells. These data provide additional evidence for a causal relationship between *HIL1* allele length anddegree of polystyrene binding.

### Human antibody responses to *D. hansenii* cell surface antigens differ between food- and patient-derived isolates

As a food-associated yeast, *D. hansenii* is frequently ingested and is readily detected in the human fecal mycobiome (8). In CD, ulcerations in the intestinal barrier lead to translocation of luminal microbes and subsequent development of systemic immune responses, including immunoglobulin G (IgG). Such antibody responses often target fungal cell wall proteins (25). Because serum antibody responses to gut microbes reflect prior and/or ongoing microbial exposure, we hypothesized that serum immunoglobulins from CD patients would bind to the cell surface components of *D. hansenii* strains, including *HIL1*. As a positive control, we included *C. albicans*, a gut-associated pathobiont that is targeted by the adaptive immune system in CD (25, 59). Using serum samples from a cohort of pediatric CD patients, we incubated yeast with serum from individual patients and detected bound IgG via flow cytometry. This assay permits characterization of IgG reactivity to exposed yeast cell surface antigens.

**Fig 5:**
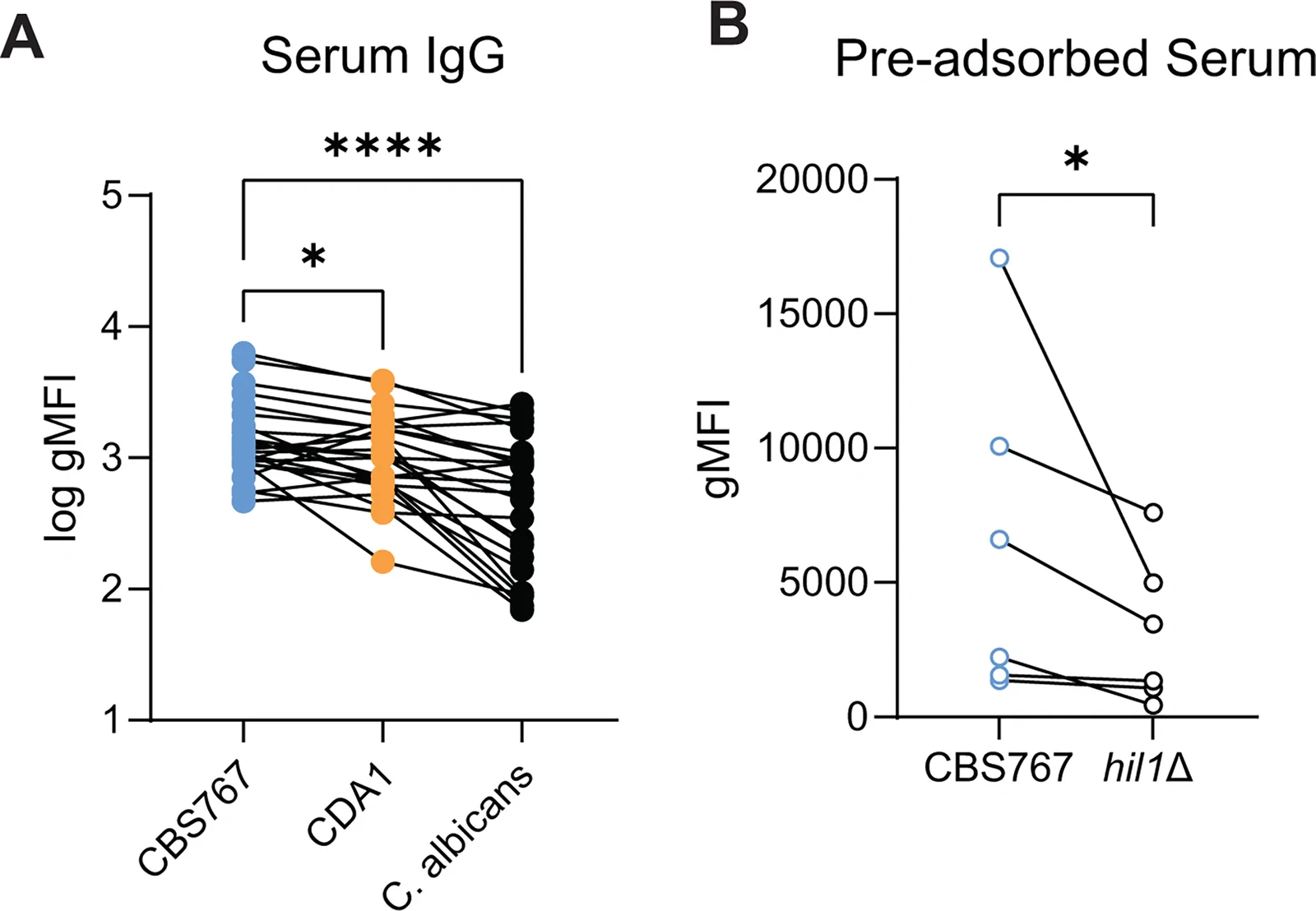
CD patient serum IgG targets *Hil1.* (**A**) Human IgG deposition on indicated *D. hansenii* (CBS767 or CDA1) or *C. albicans* (SC5314) yeast cell wall surface was quantified using flow cytometry. Geometric mean fluorescence intensity (gMFI) was log transformed prior to graphing and statistical analysis. Each dot represents an individual patient, with lines connecting isolates within a patient. Five of the 29 patients exhibited no detectable signal to any of the three tested yeast strains and were excluded from the analysis, see Materials and Methods. (**B**) Human IgG deposition on yeast cell surface was quantified using flow cytometry after serum pre-adsorption with 1 × 10^8^ cells of CBS767 *hil1*Δ gRNA2. Statistical analysis: * denotes *P* < 0.05, and **** denotes *P* < 0.001. Error bars, SD. (**A**) RM-one-way ANOVA for strain: F(1.367, 31.45) = 22.69, *P* < 0.0001. Dunnett’s multiple comparisons test compared to CBS767: CBS767 vs CDA1, *P* = 0.0391; CBS767 vs *C. albicans*, *P* < 0.0001. *N* = 24 CD patients. (**B**) Wilcoxon matched pairs signed rank test, *P* = 0.0312. *N* = 6 CD patients.

CD patients exhibited detectable levels of fungal reactive antibodies (Fig. S7A). Serum binding of *C. albicans* strain SC5314 was consistent with levels detected in previous studies (25, 60). Notably, we observed higher levels of serum IgG binding to CBS767 than *C. albicans* (Fig. 5A). Furthermore, on a per-patient basis, we detected increased IgG binding to the food strain CBS767 as compared to the patient strain CDA1. These findings support the model that there are functional differences between these two *D*. *hansenii* strains regarding immunoglobulin reactivity to the yeast cell surface.

Given that adhesins are targeted by antibody responses (24, 25), we tested if Hil1 could be specifically targeted by human antibodies. As the pre-adsorption protocol to enrich for Hil1 reactive antibodies requires sufficient starting material, we selected a subset of the CD patients with banked serum volumes compatible with this requirement. Patient serum was incubated with excess CBS767 *hil1*Δ cells to deplete antibodies that bind epitopes on CBS767 except for Hil1. As expected, we validated that pre-adsorption reduced antibody binding capacity to CBS767 relative to unadsorbed serum (Fig. S7B). Notably, pre-adsorbed serum retained binding to CBS767 WT cells to a higher level than CBS767 *hil1*Δ (Fig. 5B). This is proof of concept that CBS767 Hil1 can be targeted by human immunoglobulins. We next tested unadsorbed serum from all patients for the ability to bind CBS767 or CBS767 *hil1*Δ. We observed a reduction of antibody binding in CBS767 *hil1*Δ relative to the WT CBS767 strain (Fig. S7C). Taken together, these data indicate that for a subset of CD patients, CBS767 reactive antibodies can bind to Hil1 on the yeast cell surface.

### Hil1 protein modeling

**Fig 6:**
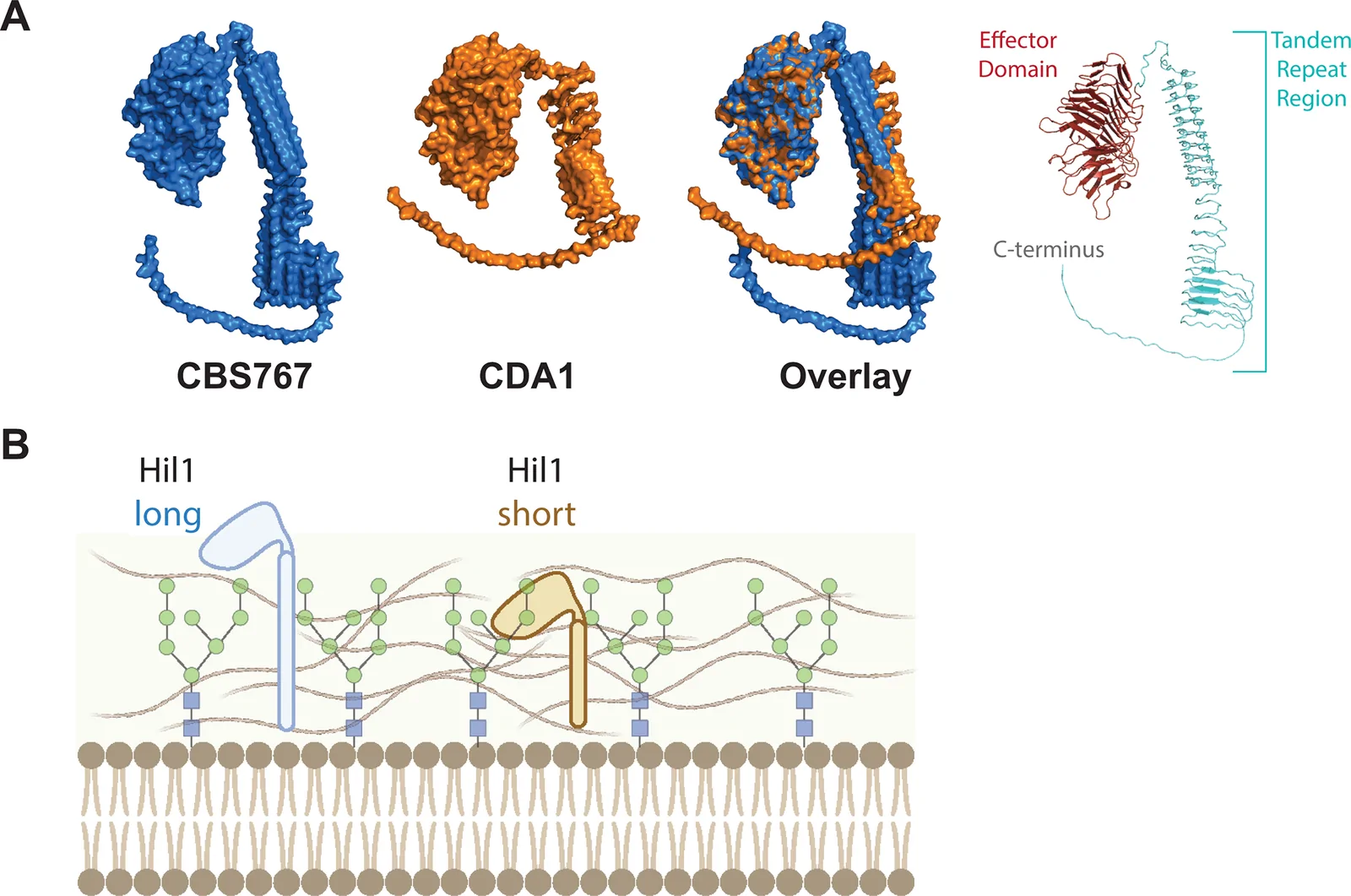
Model of *HIL1* long and short alleles. (**A**) AlphaFold3-generated surface models of the mature peptide sequences of CBS767 (blue) and CDA1 (orange). Peptides were overlaid to show homology of the effector domain. Far right, cartoon model of CBS767 allele with the effector domain labeled in red and tandem repeat region in teal. CDA1 was predicted to contain a shortened tandem repeat region or “stalk.” (**B**) Schematic of the working model depicting long and short alleles of *HIL1* of *D. hansenii* in relation to the cell wall components.

Given the extensive allelic variation in *HIL1* between *D. hansenii* strains, we evaluated the effect on predicted peptide structure using the *in silico* modeling tools. Hil1 amino acid sequences from CBS767 and CDA1 were analyzed with NetGPI and SignalP to identify predicted cleavage sites for the mature peptide (47, 61). The folding of mature amino acid sequences for CBS767 (residues 21–753) and CDA1 (residues 21–562) was predicted using AlphaFold 3 (Fig. 6A) (62). Overlaying the CBS767 allele with the CDA1 allele highlighted that the protein stalk of Hil1 is shorter in CDA1. We propose that the long alleles of *HIL1*, such as those in CBS767 and CCFD2, mediate adhesion by their ability to extend past the cell wall, while shorter *HIL1* alleles fail to extend past the glycans to mediate binding (Fig. 6B). This model is consistent with the glycan shielding observed in the short *IFF* paralogs in *C. albicans* (63).

## DISCUSSION

Our goal was to understand the phenotypic diversity of *D. hansenii* in the context of food strains that are considered to be safe for human consumption, and the existence of strains that have been isolated from patients with CD (10). Here, we show that representative food and patient strains of *D. hansenii* exhibit a spectrum of phenotypic diversity related to adhesion and biofilm formation. Using loss-of-function forward genetic screening and CRISPR-Cas9 reverse genetic validation, we found a single adhesin, Hil1, which governed adhesion-relevant phenotypes in a model food strain. Comparative genomic analysis of this adhesin revealed polymorphisms in the tandem repeat domain between strains that correlated with the degree of adhesion and biofilm-relevant phenotypes. Using a heterologous expression system to test function, we showed that the shortened *HIL1* allele from the CDA1 patient strain failed to confer an adhesion phenotype, while the longer CBS767 allele was able to do so. Furthermore, we show that Hil1 is targeted by serum immunoglobulin in CD patients. This binding is reduced in a strain of *D. hansenii* with an engineered loss of *HIL1*. In summary, we show that genetic tractability and comparative genomics enabled the identification of a microbial mechanism that dictated CD-relevant host responses to different strains of *D. hansenii*.

The Hil1 structure/function relationship is dictated by alterations in the tandem repeat number within the coding sequence of *HIL1. D. hansenii* strains encoding shortened tandem repeat domains in Hil1 were less adherent, despite conservation of the adhesin effector domain. Furthermore, heterologous expression of a long (*HIL1^CBS767^*) but not short (*HIL1^CDA1^*) allele rescued polystyrene binding in *S. cerevisiae*. Alterations to tandem repeat copy number are a canonical mechanism of genetic diversity across kingdoms (64). Intragenic repeat alterations have been proposed as a mechanism of rapid adaptation to the environment for yeasts, including evasion of the host immune response through cell surface antigen variation (43). In *S. cerevisiae*, pellicle formation is directly correlated with *FLO1* intragenic tandem repeat length, akin to our observations of *D. hansenii HIL1* (43). In *C. albicans*, shorter *IFF* paralogs like IFF8 fail to extend past the outer limits of the cell wall glycans, limiting antibody-mediated detection through steric interactions (63). Our conceptual model, informed by our understanding of adhesins across many yeast species, posits that shortened tandem repeat domains, which function as the “stalk” of the protein, reduce the capacity of the adhesin domain to be exposed past the cell wall glycans and thereby block adhesion (Fig. 6A and B) (63). This model of glycan shielding provides a rational explanation for how the loss of *HIL1* expression phenocopies expression of shortened HIL1 alleles; in shortened Hil1, the functional effector domain may be unable to interact with the environment to mediate adhesion. Furthermore, this model is consistent with the increased CD serum antibody binding of the long Hil1 cell wall protein in CBS767.

Adhesins from multiple microbial kingdoms are considered factors that facilitate infection by mediating adherence to host surfaces. Known yeast pathogens contain, on average, over ten *HIL* paralogs, while *D. hansenii* contains only three (19, 21). Specialization of the multiple adhesins in the genome of pathogens, such as *C. auris*, permits loss of function of single members affecting phenotypes and adhesion to specific surfaces and cell types (18, 35). We validated the expression of three *HIL* paralogs in the *D. hansenii* food strain CBS767, consistent with the notion that less pathogenic yeasts contain fewer adhesins than pathogenic yeast species (19, 21). Thus, it was surprising that the patient isolated CDA1 has a complete genetic loss of *HIL3* and transcriptional suppression of *HIL2*. Furthermore, the *HIL1* allele present in CDA1 failed to confer adhesion in a heterologous expression system, indicating the allele may be defective. The advantage of these adaptations to a yeast located within a CD intestinal ulcer is unknown, but we suggest a few possibilities. First, the ability for yeast to form biofilm can lead to expulsion of greater numbers of cells due to their connectivity. Patient strains of *D. hansenii* that do not form biofilms would thus be more difficult to expel by this mechanism. Second, the adaptive immune system targets adhesins by the production of neutralizing antibodies. A patient strain of *D. hansenii* that lacks targetable antigen would potentially evade this effect. For the latter point, *C. albicans’* commensalism is shaped by neutralizing antibodies targeting adhesins (25). Others have also posited that rapid variation in intragenic tandem repeats may be a mechanism of cell wall antigen variation and immune evasion by pathogens (43). It is therefore plausible that loss of adhesive phenotypes in *D. hansenii* CD isolates may be related to potential immune evasion.

While the *D. hansenii* Hil adhesins contain structural orthologs in related CTG clade fungi, the genomic context of these adhesins is unique to *D. hansenii*. Synteny analysis of related CTG clade yeasts, such as *Debaryomyces fabryi*, *C. albicans*, *Candida tropicalis*, and *C. auris*, shows a lack of genomic orthology. For *HIL1* specifically, it is one of eight terminal ORFs in Chromosome G of *D. hansenii* that are differentially present with related species *D. fabryi*. The location of *HIL1*, and of *HIL3*, both located within ~20 kb of the end of the chromosome, is consistent with subtelomeric enrichment of other adhesins (21, 42). The lack of synteny is likely a feature of their subtelomeric enrichment, as these loci are known for their ability to undergo rapid diversification between closely related species (65).

Although we provide evidence here that the *HIL1* allelic variation affects polystyrene adhesion phenotypes, we note that modifiers may be present between strains of *D. hansenii* that may be extrinsic to *HIL1*. For instance, patient strain CDA1 appears to be deficient in *HIL3* and exhibits reduced expression of *HIL2*; future studies generating the multiplicity of knockouts of *HIL1, HIL2,* and *HIL3*, alone, and in combination, alongside complementation, will be necessary to parse out each *HIL* family member’s relative contributions to adhesion. Additionally, while mapping our AtMT mutants, we observed rare cases of naturally occurring loss of *HIL1* through subtelomeric deletions that appeared to be independent of t-DNA insertions. Future studies detailing the rate of genomic instability at this locus, including intragenic *HIL1* tandem repeat contraction/expansion, or subtelomeric deletions, or translocations, would be of interest for understanding the mechanisms that give rise to the genomic diversity of *D. hansenii* strains.

An important takeaway of our study is that the genetic and phenotypic diversity between *D. hansenii* strains is not limited by isolation source: there exist strains of *D. hansenii* in the food supply that phenocopy features of isolates recovered from CD patient ulcers. Other studies have suggested that food-associated fungi can exacerbate colitis (66). Given that ingested food-derived fungi are a common source of fungal reads in fecal microbiome sequencing (1), it is plausible that in CD patients with barrier defects due to disease, ingested food strains of *D. hansenii* may cross the barrier and result in adaptive immune responses. This is supported by the fact that IBD patients commonly exhibit antibodies that target *S. cerevisiae* (67), and is consistent with our data showing that Hil1 is a target of serum IgG from CD patients.

## MATERIALS AND METHODS

### Strains and culture conditions

The *D. hansenii* reference strain CBS767 was obtained from ATCC (#36239). Crohn disease isolates CDA1, CDA2, and CDE1 were described elsewhere (10), and the cheese strains (DH242, DH246, DH60, DH76) were published previously (12). Cheese strains (CCFD1–CCFD4) were cultured for the present study at the Cleveland Clinic from commercially available cheeses as described elsewhere (56). Briefly, ~50 mg of the cheese sample was added to 450 µL of sterile PBS and vortexed for 15 s to create a slurry. Tenfold serial dilutions were plated on Sabouraud’s Dextrose Agar containing 50 μg/mL of chloramphenicol and incubated at room temperature until colonies developed. Strains were validated as *D. hansenii* by Sanger sequencing of the ITS1-5.8SrRNA-ITS2 locus (58) and discriminatory PCR of the PAD1 locus (57). *S. cerevisiae* strain BY4739 (S288C background; MATα *leu2*Δ *lys2*Δ *ura3*Δ) was a gift from Kurt Runge (Cleveland Clinic). Strains were routinely cultured on YPD agar plates or liquid medium without antibiotics at 30°C, except for CCFD4, which was grown at room temperature. For pellicle quantification, stationary phase cultures were placed vertically in a tube rack undisturbed at room temperature for at least 24 h to allow for pellicle formation. For colony morphology analysis, cells were revived from glycerol stocks onto YPD medium and photographed after 2–3 days in culture at 30°C.

### Crystal violet biofilm quantification assay

Quantification of biofilm formation was conducted as previously described (44). Briefly, overnight cultures of *D. hansenii* strains were back diluted to an optical density of 600 nm (OD_600_) of 0.15 in fresh YPD in 1 mL aliquots. Of this stock, 100 μL was added to a non-tissue culture-treated polystyrene round-bottom 96-well plate (Corning, Falcon, #351177) and allowed to sit at room temperature undisturbed for 24 h to adhere to the plate. Where indicated, tissue culture-treated polystyrene plates were also used (Corning, Costar, #3799). Plates were flicked into a waste bucket, submerged face up in a bucket of distilled water, and flicked into the waste bucket again. This was repeated for two total washes. Washed wells were stained with 125 μL of 0.1% crystal violet solution in water for 15 min. Plates were similarly washed again for four total washes and allowed to dry completely. Deposited crystal violet on the plate was solubilized with 125 μL of 30% acetic acid for 10 min. From this, 100 μL were transferred to a flat-bottom 96-well plate, and absorbance at OD_550_ was read. The background was subtracted from control wells containing YPD alone. Each data point is the average of two to three technical replicates per experiment.

### *Agrobacterium tumefaciens*-mediated transformation

The CTG clade-adapted AtMT system was a gift from Teresa O’Meara (45). *A. tumefaciens* strain pTO132, which is the EHA105 strain of *A. tumefaciens* transformed with pTO128 nourseothricin resistance T-DNA plasmid, was cultured on LB with 50 μg/mL of kanamycin at 30°C, and single colonies were grown in LB with kanamycin at 30°C, shaking at 240 rpm. Overnight liquid cultures were washed and resuspended in 5 mL of induction medium IM [1× MM salts, 10 mM glucose, 0.5% glycerol, 2-(N-morpholino)ethanesulfonic acid (MES) 7.7 g/L, acetosyringone 3′,5′-dimethoxy-4-hydroxyacetophenone 19 mg/L, pH 5.3] acetosyringone and cultured for 7 h at room temperature by shaking 240 rpm in a 15 mL round bottom tube to a final OD_600_ of around 0.6. Overnight cultures of *D. hansenii* were washed in sterile water, counted, and diluted to 1 × 10^7^ cells/mL in water. An *A. tumefaciens* suspension (OD_600_ of 0.6 to 0.75) and yeast were mixed 1:4 and plated in two 200 µL droplets onto IM agar plates (MM salts, 5 mM glucose, 0.5% glycerol, 2% agar, MES 7.7 g/L, acetosyringone 20 mg/L, pH 5.3) and cocultured for 4 days at room temperature protected from light. Plates were scraped with 5 mL of YPD and an L-shaped plastic spreader to recover cells. Yeast was recovered by centrifugation at 1,000 × *g* and washed two more times with 5 mL of YPD to enrich for fungal cells. Cells were resuspended in 1 mL of YPD, and 100 μL was spread onto YPD agar plates with 200 μg/mL of nourseothricin and 100 μg/mL cefotaxime. After 4 days of growth on selective agar, colonies were visually screened using a dissection microscope, and convertants to Sm morphology were selected. Single colonies were picked with a sterile toothpick into 96-well barcoded plates (Costar 3904BC) with 100 μL of YPD. Plates were grown shaking at RT at 150 rpm overnight, OD_600_ was read to confirm growth, and then overlaid with 100 μL of 40% glycerol in water, triturated, and frozen at −80°C. Concentration of 1× MM salts: KH_2_PO_4_ 1.5 g/L, K_2_HPO_4_ 2 g/L, NaCl 0.15 g/L, MgSO_4_·7H_2_O 0.5 g/L, CaCl_2_·2H_2_O 66 mg/L, FeSO_4_·7H_2_O 2.5 mg/L, (NH_4_)_2_SO_4_ 0.5 g/L.

### Insertional mutagenesis mapping

Genomic DNA was isolated from 12 insertional mutants using the MasterPure Yeast DNA purification kit (LGC, MPY80200) and quantitated using the Quant-iT dsDNA broad range assay (ThermoFisher Q33267). DNA from three mutants was pooled ratiometrically for whole-genome sequencing. Samples were sequenced by SeqCoast Genomics (Portsmouth, NH, USA) at a depth of ~28× coverage for each 12 Mbp genome. Libraries were prepared using the Illumina DNA Prep tagmentation kit (#20060059) with Illumina Unique Dual Indexes. Sequencing was performed on the Illumina NextSeq 2000 platform using a 300-cycle flow cell kit to produce 150 bp paired-end reads. Read demultiplexing, read trimming, and run analytics were performed using DRAGEN v4.2.7. Mapping of the t-DNA insertion site was performed as previously described (45). In brief, genomic reads were analyzed using Galaxy (UseGalaxy.org) (68). Adaptors were trimmed using Cutadapt. Sequences were aligned to the t-DNA insertion sequence using BWA-MEM alignment to the t-DNA insertion sequence with a minimum seed length of 50 and a bandwidth of 2. BAM files were opened using GenVision Pro (DNASTAR Lasergene 17). Reads that mapped within 10 bp of the t-DNA borders were selected and assembled into contigs using SeqMan Ultra with 90% alignment cutoff (DNASTAR Lasergene 17). BLAST was used to identify the incorporation location of each contig within the *D. hansenii* genome. Primers were designed to identify inserts, and pools were deconvoluted with PCR amplification of the mapped insertion site.

### Sequencing and genome assembly of *D. hansenii* isolates and identification of *HIL1* alleles

Strains were grown in YPD at room temperature, and genomic DNA was isolated using a Zymo ZR Fungal/Bacterial DNA Miniprep kit or LGC MasterPure Yeast DNA Purification Kit (MPY80200) according to the manufacturer’s instructions. Strains were sequenced by Illumina (NextSeq2000 platform using a 300-cycle flow cell kit to produce 2 × 150 bp paired reads, using Illumina DNA Prep tagmentation kit and Illumina Unique Dual Indexes) with SeqCoast Genomics (Portsmouth, NH, USA); a sequencing threshold of 400 Mbp/2.7 million reads, which was estimated to provide 30× final coverage. Read demultiplexing, read trimming, and run analytics were performed using DRAGEN (versions 3.10.12 or 4.2.7). *De novo* genome assemblies were generated from short-read data for the experimental strains without public genome assemblies. CBS767 was assembled as a methods control using SPAdes v3.15.5. Prior to assembly, all short and long reads were evaluated by FastQC (69).

Local BLAST (blastn) was performed against each *de novo* Illumina genome assembly for each strain, using the *HIL1* nucleotide sequence from the CBS767 RefSeq genome as the query sequence. DNA sequences for this locus were extracted from each strain’s genome assembly using blastn match coordinates in R. The DEHA2g2490g/*HIL1* gene was identified in all strains except CCFD4. FASTA files for genes and amino acid sequences were generated. All genes remained in frame despite varying tandem repeat numbers. Multi-sequence alignment of amino acid sequences was performed using MegAlign software (DNASTAR Lasergene 17).

### HIL1 repair cassette generation

Plasmids were a kind gift from Nicholas Papon (70). Plasmid pAYCU268, which contains the nourseothricin resistance gene *SAT1* under control of the *Candida dubliniensis TEF1* promoter, was used as a backbone. The *D. hansenii TEF1* promoter was swapped in from CBS767 as previously described (70) upstream of the antimicrobial resistance cassette in pAYCU268 to generate pKN403 (P_*Dh*_*TEF1–SAT1–T_Mg_PGK1*; P_*Mg*_*ACT1–yeGFP–T_Mg_TRP1*; *f1 ori*; *AmpR*). Plasmid sequences were verified using long-read whole-plasmid sequencing (Eurofins Genomics). Primers (listed in Table S3) were designed to create an HDR cassette where P_*Dh*_*TEF1–SAT1–T_Mg_PGK1* was flanked by roughly ~500 bp of the promoter and terminator of CBS767 *HIL1* (30). In brief, PCR amplification of the left flank and right flanks contained ~20 bp of overlapping homology with primers amplifying the pKN403 P_*Dh*_*TEF1–SAT1–T_Mg_PGK1* sequence. NEB HiFi assembly master mix (#E2621) was used to anneal the three fragments. A second set of primers nested within the start and end of the cassette was used to amplify the full cassette for PCR purification with Primestar Max V2 (Takara) polymerase. Cassettes were purified using the Monarch PCR Cleanup kit (NEB) and eluted in water.

### Preparation of Cas9 RNP complexes

CRISPR Cas9 RNP complex components were synthesized by IDT (Alt-R System). Synthetic guide RNAs (sgRNAs) were designed using the IDT gRNA design tool using the CBS767 reference genome-derived gene sequences and validated for specificity and off-target effects using EuPaGDT (48). Per manufacturer directions, CRISPR RNA was duplexed with tracrRNA to form sgRNAs, and RNPs were formed with 3 μL of 6 μM Cas9 protein (IDT 1081060) with 3.6 μL of sgRNAs. RNP complexes (6.6 μL) were added to the cell slurry prior to electroporation as described below.

### Transformation of *D. hansenii*

To prepare electrocompetent *D. hansenii* cells, the indicated strains of *D. hansenii* were grown to an OD_600_ of 1–2 in 50 mL of YPD medium. Cells were pelleted, washed with distilled water, and added to a flask with 10 mM Tris, 1 mM EDTA, pH 8.0, and 0.1 M lithium acetate and shaken at 150 rpm for 1 h at room temperature. Dithiothreitol was added to a final concentration of 25 mM and shaken at room temperature for 30 min. Cells were harvested via centrifugation and washed twice with ice-cold distilled water and resuspended in ice-cold 1 M sorbitol to form a cell slurry. A total of 1.5–2 μg of HDR repair cassette was mixed with 40 μL of competent cell slurry in sorbitol with or without prepared RNPs and set on ice for at least 5 min. Samples were electroporated in a 0.2 cm electroporation cuvette with Biorad GenePulser X-Cell at 2,300 V, 25 μF, and 100 Ω. Cuvettes were flooded with 1 M ice-cold sorbitol, pelleted via centrifugation, and resuspended in 1 mL of YPD + 100 mM sorbitol for 2 h of static recovery. Four milliliters of YPD + 100 mM sorbitol were then added and shaken overnight at room temperature to allow for outgrowth prior to selection. Cells were plated on YPD agar containing 200 μg/mL of nourseothricin and allowed to grow for 4 days at room temperature for colonies to form.

### Fungal gene expression analysis

Indicated yeast cells were cultured overnight at room temperature in YPD medium, shaking at 240 rpm. A volume of 250–1 mL of dense culture was pelleted and RNA was isolated using the MasterPure yeast RNA purification kit (Lucigen, MPY03100) per manufacturer’s directions. RNA was quantitated using a NanoQuant plate (Tecan). One microgram of total RNA was added to a 20 μL cDNA synthesis reaction (iScript, BioRad). For No-RT control, the same quantity of RNA was diluted into water. For each 25 μL PCR reaction, 22.5 ng of cDNA or equivalent no-RT control was amplified using ApexRed Taq (Genesee Scientific). The resulting PCR product was run on a 2% Tris-Borate-EDTA gel to resolve products. Primers used are listed in Table S3. *D. hansenii ACT1* or *S. cerevisiae TAF10* (71) were used as reference controls. Proposed names and their associated genes present in the CBS767 reference genome are as follows: *HIL1* (DEHA2G24970g), *HIL2* (DEHA2B01452g), *HIL3* (DEHA2D00132g), and *ACT1* (DEHA2D05412g).

### Growth curves

Indicated strains were grown overnight, shaking at 30°C, before back dilution in fresh YPD to an OD 0.05. One hundred microliters were added to a flat-bottom tissue culture-treated 96-well plate (Falcon). Plates were grown at 30°C in Cyation5 (Cytek), and OD_600_ was read every 15 min for 24 h. Plates were shaken continuously in an orbital pattern at a frequency of 807 cpm (1 mm). Growth rate was calculated using the R package GrowthCurvR (72).

### Cell spreading and colony biofilm

YPD medium was prepared as either semisolid or regular agar concentrations (1% yeast extract, 2% peptone, and either 0.5% or 1.5% agar) and autoclaved at 121°C for 15 min. Dextrose was autoclaved separately and added to a final concentration of 2%. Agar was cooled to 60°C before pouring 25 mL plates. Plates were dried for 90 min with the lids ajar prior to inoculation.

For plate inoculation, overnight cultures were grown in liquid YPD at room temperature (RT) on a roller drum. Cells were washed three times with sterile water, resuspended to an OD_600_ of 1 in 200 μL of sterile water, and then inoculated (5 μL) onto each plate. Once the inoculum was dry, and 0 h colony diameter was measured, they were incubated in a tray lined with wet paper towels and covered with a plastic film to keep the plates hydrated. Plates were incubated at 30°C for 48 h. After 48 h, colonies were measured, then resuspended in 500 μL of sterile water, and vortexed for 10 s, and the OD_600_ was measured in microtiter plates in a spectrophotometer.

### Cell surface hydrophobicity

CSH was measured as described previously (51). Overnight cultures (5 mL) grown in YPD were washed two times using sterile water and resuspended in 6 mL at 0.2–0.6 OD_600_ in glass test tubes. After an initial absorbance measurement at 600 nm (*A*0) using a Spec20, 800 µL of n-hexadecane was added. Tubes were sealed with plastic film, vortexed for 30 s, then allowed to stand at 30°C for 2 min prior to a second absorbance measurement (*A*1). CSH was calculated using the following formula: [1 − (*A*1/*A*0) × 100.

### Heterologous expression of *D. hansenii HIL1* in *S. cerevisiae*

*HIL1* alleles from CBS767 or CDA1 were cloned from genomic DNA. Plasmids were assembled, including the *GAL* promoter from *S. cerevisiae*, the indicated *HIL1* allele, *the ADH1* terminator, a natMX6 selectable marker, and a CEN/ARS sequence and plasmid backbone from the yeast_DualReporter plasmid (gift of Aviv Regev, Addgene plasmid # 127546; http://n2t.net/addgene:127546; RRID:Addgene_127546) (73) using NEB HiFi assembly (E2621S). Electrocompetent BY4739 *S. cerevisiae* cells were generated as described above with *D. hansenii* with the following modifications: electrocompetent cells were electroporated with 0.2 μg of plasmid at 1,500 V, 25 μF, 200 Ω, in a 0.2 cm cuvette. Cells were outgrown for 2 h at 30°C, shaking at 240 rpm, before plating on YPD plates supplemented with 100 μg/mL of nourseothricin. Three independent transformants per group were streaked onto YP-2% raffinose plates with nourseothricin. Single colonies were grown overnight in YP-2% raffinose medium with 100 μg/mL of nourseothricin at 30°C, shaking at 240 rpm. Wild-type BY4739 cells were grown without nourseothricin as a control. Cells were back-diluted to an OD_600_ of 0.2 in fresh YP-2% raffinose-2% galactose and nourseothricin and grown for 5 h to mid-log (OD_600_ of 0.4–0.6). Cells were then back-diluted into fresh YP-2% raffinose-2% galactose with nourseothricin selection as appropriate at an OD_600_ of 0.15. Cells were plated in 96-well round-bottom polystyrene (non-tissue culture treated) plates for 24 h to measure biofilm formation. Crystal violet staining was performed as described above.

### Crohn disease serum samples

Banked serum samples from patients recruited under the Institutional Review Board Student 21-1205 at the Cleveland Clinic Foundation were used to determine serum IgG binding of yeast strains. Patients aged 6–17 undergoing standard-of-care colonoscopy for evaluation of Crohn disease were recruited for the study. Informed consent was provided by parents/guardians of patients aged 6–17 years, and assent was obtained from children aged 7–17 years to participate in the study. Blood was collected in a gold top serum separator tube and centrifuged at 1,350 *× g* for 15 min at 4°C to recover serum. Serum from each patient was frozen in multiple aliquots and stored at −80°C until analysis. Of the 30 Crohn disease patients initially recruited, 29 had remaining serum banked for the present analyses. Demographic information for these 29 patients includes an average age of 13.97 ± 3.08 years, 38% female gender, and 93% white race.

### Cell surface immunoglobulin binding assay

Immunoglobulin flow cytometry was performed to test the binding of human serum antibody to *D. hansenii* as described in reference 25. Yeast strains were cultured overnight in YPD at either 30°C (Fig. 5A, CBS767, CDA1, or SC5314) or room temperature (Fig. S7C, CBS767, *hil*1∆KO1, *hil*1∆KO2). Cultures were washed and normalized to an OD_600_ of 0.5 in P/B/A (PBS, 1% bovine serum albumin, 0.01% sodium azide), counted using a hemocytometer, and diluted to 2 × 10^7^ cells/mL. Human serum was diluted 1:75 in PBS. Twenty-five microliters of each strain (5 × 10^6^ cells) and 25 µL of diluted serum were plated in technical triplicate in a TC-treated 96-well v-bottom plate (Corning #3894) and co-incubated on ice for 45 min. Cells were washed twice via centrifugation at 3,000 rpm for 5 min at 4°C with 150 μL of P/B/A. After washing, cells were stained with 50 μL of donkey anti-human IgG PE antibody (Jackson Immuno 709-116-149) diluted 1:500 in column buffer (PBS, 2 mM EDTA, 10 mM HEPES, 0.5% FBS) and incubated on ice in the dark for 20 min. Cells were washed twice in column buffer, then transferred to a TC-treated round-bottom 96-well plate for flow cytometry analysis using the Attune NXT with autosampler (ThermoFisher). Unstained yeast and no-serum controls were used to identify the gating for IgG-positive cells. Geometric mean fluorescent intensity (gMFI) of IgG binding was calculated using FlowJo (BD v 10.9.0). Data were normalized by log transformation and analyzed using GraphPad Prism 10. Our initial comparison (Fig. 5A, CBS767, CDA1, or SC5314) found that 5 of 29 patients exhibited no binding to any of the three tested yeast strains, defined as <3% positive events within the IgG-PE+ gate relative to the secondary antibody-only stained negative control, and were excluded from the analysis and downstream experiments.

### Pre-adsorption of serum with *hil1*Δ *D. hansenii* cells

To enrich for Hil1 reactive antibodies, CBS767 *hil1*Δ gRNA2 clone was cultured overnight in YPD at room temperature, washed, resuspended to an OD_600_ of 0.5 in P/B/A, and counted using a hemocytometer. A total of 1 × 10^8^ yeast cells were pelleted by centrifugation at 3,000 rpm at 4°C for 5 min, and the supernatant was discarded. The yeast pellet was resuspended in 200 µL of normal human serum with 1× protease/phosphatase inhibitor (CST #5872S). Six patients were selected who had banked serum volumes compatible with this protocol. Serum was incubated with yeast for 45 min at 4°C with end-over-end rotation to mix. Yeast was removed by centrifugation at 2,000 *× g* for 5 min at 4°C. A volume of 150 µL of each serum sample was further clarified by another round of centrifugation at 2,000 *× g* for 5 min at 4°C. Prior to the binding assay, adsorbed serum was diluted 1:2 in PBS and clarified a final time by centrifugation at 10,000 × *g* for 1 min at 4°C. The assay was performed using the same methods as described previously, using donkey anti-human IgG PE at a 1:250 dilution.

### Modeling of Hil1 protein structure

Amino acid sequences were generated from *HIL1* genes identified as described above. GPI anchor site attachment and signal peptide cleavage sites were predicted using NetGPI V1.1 and SignalP V6 peptide (47, 61). SignalP analysis predicted with high confidence (likelihood 0.9996) that HIL1 contained a signal peptide, with a probability of 0.980142 that the cleavage site was between amino acids 20 and 21. NetGPI predicted that amino acid S753 (CBS767) or S562 (CDA1) was the omega site for conjugation to the cell wall with a likelihood of 0.507. Mature peptide sequence folding for CBS767 (amino acid residues 21–753) or CDA1 (21–562) was predicted by AlphaFold 3 (62) and visualized using the PyMOL molecular graphics system, version 3.1 (Schrödinger, LLC). A conceptual model was created in BioRender (T. Stappenbeck, 2026, https://BioRender.com/s2667sg).

### Glycosyl linkage analysis

Glycosyl linkage analysis was performed by combined gas chromatography-mass spectrometry of the partially methylated alditol acetate (PMAA) derivatives produced from the samples as described previously (74). Briefly, permethylation of the sample was achieved by two rounds of treatment with sodium hydroxide (15 min) and methyl iodide (30 min). The sample was then hydrolyzed using 2 M TFA (2 h in a sealed tube at 120°C), reduced with NaBD4, and acetylated using acetic anhydride/pyridine. The resulting PMAAs were analyzed on an Agilent 7890A GC interfaced to a 5975C MSD (mass selective detector, electron impact ionization mode); separation of the neutral sugars was performed on a 30 m Supelco SP-2331 bonded phase fused silica capillary column. For visualization of the amino residues, the samples were run using a less polar EC-1 bonded phase fused silica capillary column.

## Data Availability

Please direct to the corresponding authors requests for strains or materials used in this study, which will be made available through means of material transfer agreement. Data used to generate figures are deposited to Zenodo (75). Whole-genome sequencing data for *D. hansenii* strains have been deposited at the Sequenced Read Archive (Bioproject PRJNA1368883).

## SUPPLEMENTAL MATERIAL

Supplemental Material mbio.01107-26-s0001.pdf Figures S1 to S7 and Tables S1 to S3.

ASM does not own the copyrights to Supplemental Material that may be linked to, or accessed through, an article. The authors have granted ASM a non-exclusive, world-wide license to publish the Supplemental Material files. Please contact the corresponding author directly for reuse.
