# Supplementary material for: The role of HIL1 in strain-level adhesion and immune recognition in Debaryomyces hansenii
Source: mBio. 2026 Jul 30;17(9):e01107-26. doi: 10.1128/mbio.01107-26 (PMC13556252; doi:10.1128/mbio.01107-26)
Supplement: Supplementary file 1 [file mbio.01107-26-s0001.pdf]

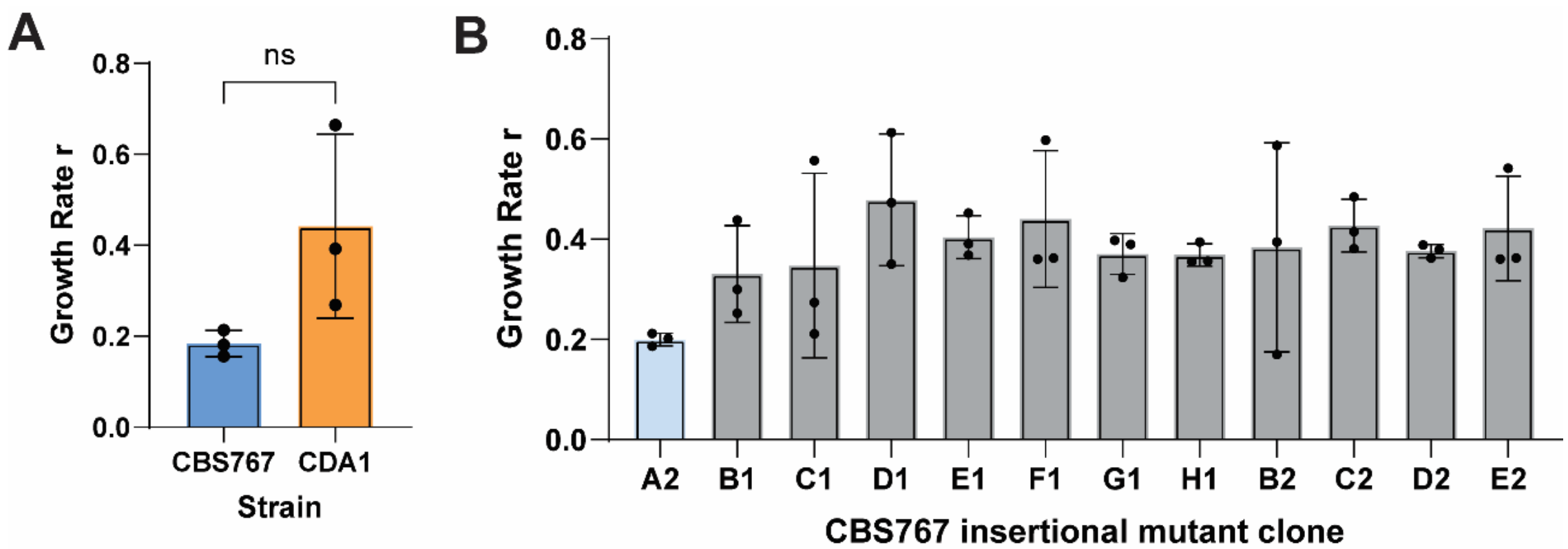

Figure S1. *Growth rate of indicated* D. hansenii *strains and mutants*. *(A,B)* Calculated growth rate of indicated strain cultured in 100 µL of YPD for 24h in Cytation5 plate reader with the OD_{600} measured every 15 min for 24 h with constant orbital rotation. *Statistical Analysis:* Error bars, SD. *(A)* Paired t-test t(2)=1.945, p=0.1912. (*B)* RM One-way ANOVA, F(11, 22)=1.485, p=0.2066.

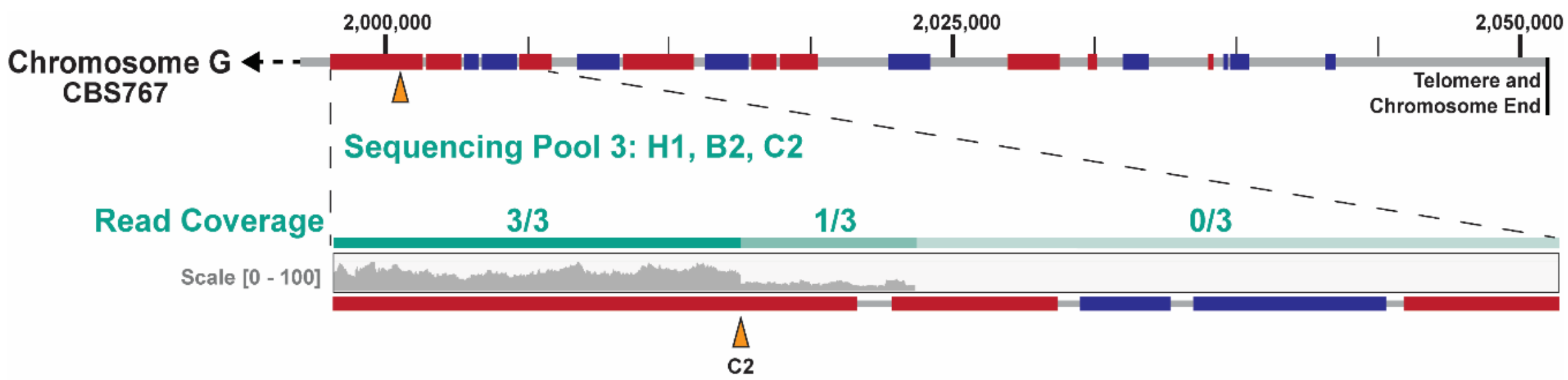

Figure S2. *Schematic of subtelomeric deletions detected in AtMT mutants.* Read coverage plots of aligned reads from a pooled sequencing reaction with AtMT mutants H1, B2, and C2 at ~28x coverage per genome. The number of reads mapping to each site is depicted in the histogram plot (gray); scale 0 to 100 reads. Teal bar highlights the relative proportions of reads at indicated regions. There were no detected reads downstream of the highlighted inset region in the mutant pool. A reduction in read coverage was observed distal to the t-DNA insertion site of C2 (orange bar). The B2 t-DNA mapped to Chromosome B, suggesting the loss of Chr G subtelomeric region was independent of the t-DNA insertion. The H1 deletion was unable to be mapped to a t-DNA insertion.

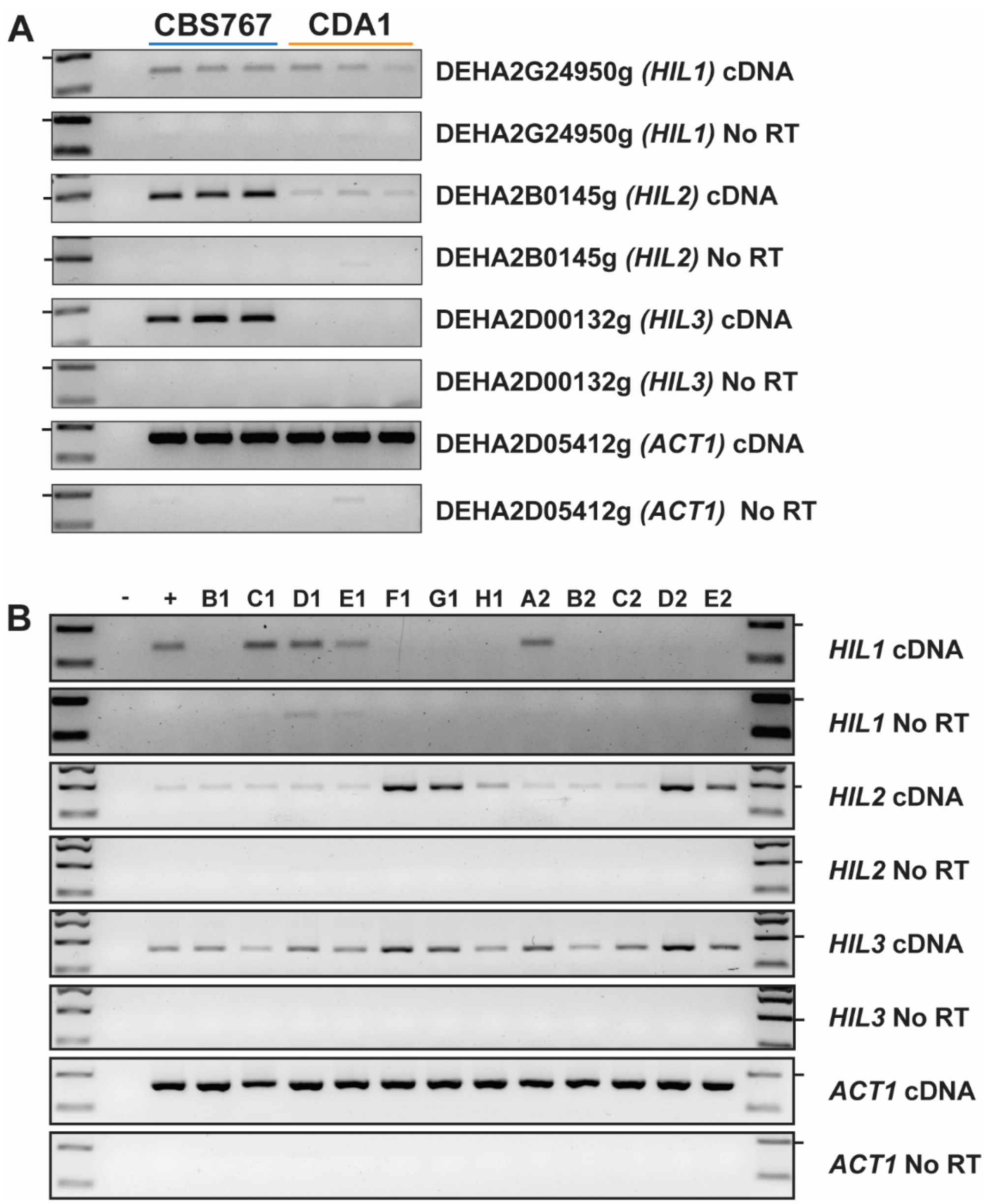

Figure S3. *Transcriptional expression of* D. hansenii *genes HIL1, HIL2, and HIL3. (A)* SYBR safe stained agarose gel of indicated endpoint Reverse Transcription (RT)-PCR products CBS767 or CDA1. Each lane represents an independent culture of indicated strain. RNA was isolated after one day of culture in YPD broth at room temperature under

constant shaking. Marker highlights 200bp ladder. *(B)* Endpoint RT-PCR analysis of indicated insertional mutant clones from positive control (+) CBS767 parental strain or indicated AtMT mutant. A2 is a Wr negative control transformant. Marker highlights 200bp ladder.

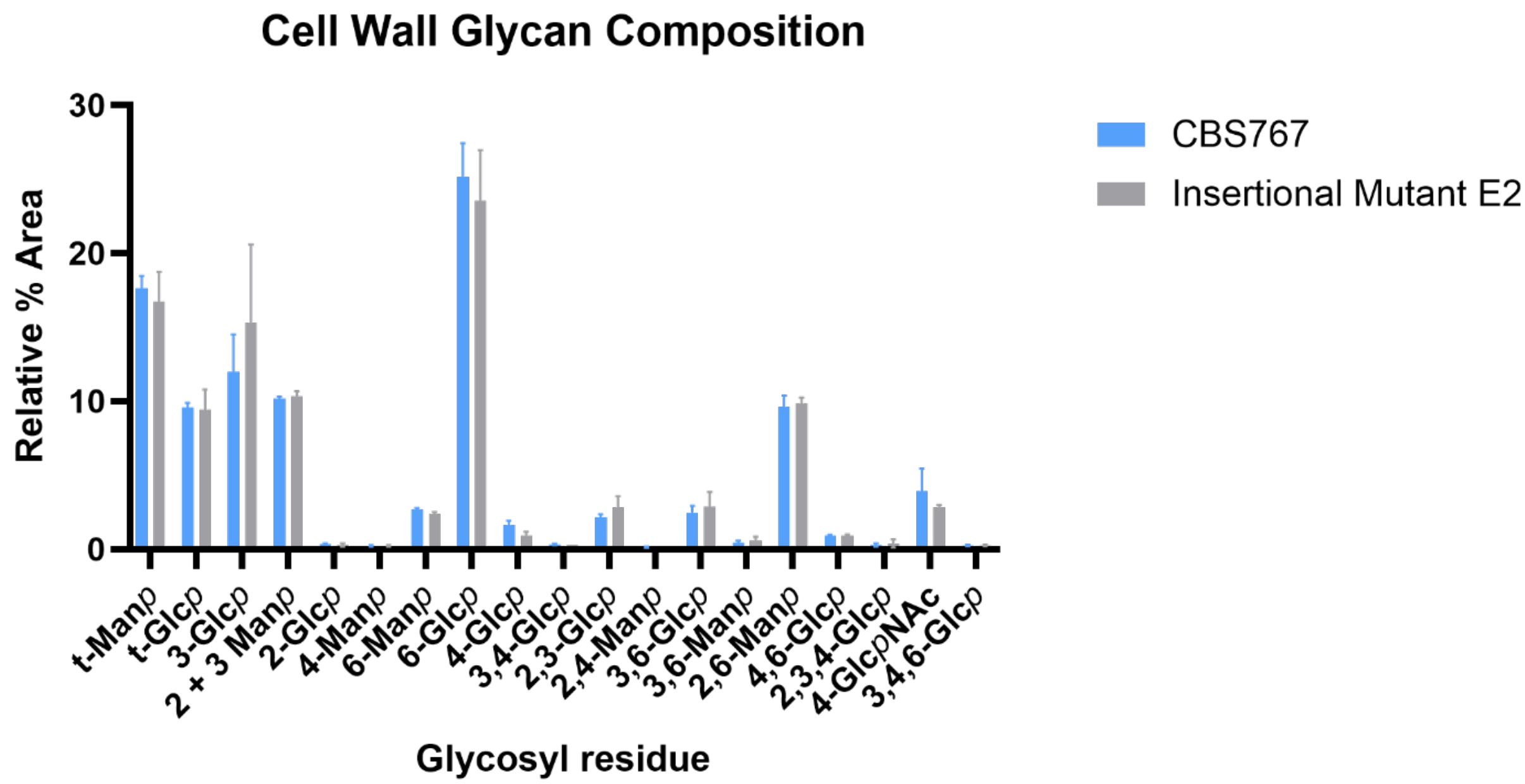

Figure S4. *No differences in cell wall glycan composition between CBS767 and AtMT Sm mutant E2*. *(A)* Gas chromatograms were measured for relative area of each species present in the yeast cell wall and identified via mass spectrometry. Data presented are the relative abundance of glycosyl species. Bars denote mean, with standard deviation. Statistical analysis conducted using multiple Mann-Whitney tests, P values corrected for multiple comparisons via FDR. All tests failed to reach threshold for a significant discovery. Error bars, SD. N=3 independent samples per strain.

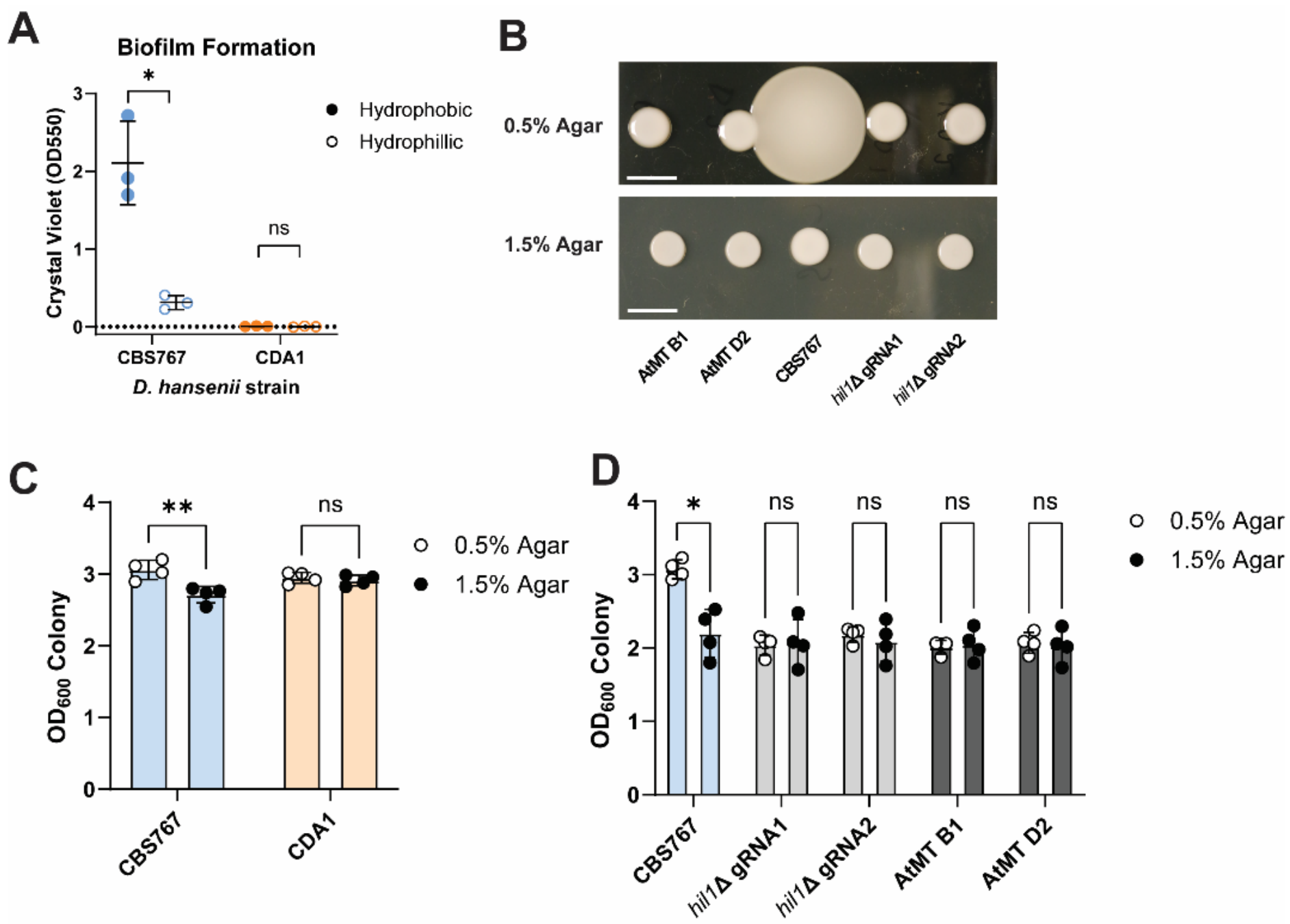

Figure S5. *Biofilm formation differences in* D. hansenii *strains on numerous substrates***.** *(A)* Quantification of 24-hour biofilm formation of indicated strains by crystal violet assay. Strains were tested using either uncoated polystyrene (hydrophobic) plates or tissue culture treated polystyrene (hydrophilic) plates. Each dot is an independent biologic replicate. B. Representative photograph of strains cultured on 0.5% or 1.5% YPD agar plates. Bar = 1 cm. *(C,D)* Quantification of scraped colony growth measured via OD_{600}. *Statistical Analysis:* * denotes P<0.05., ** denotes P<0.01, “ns” denotes non-significant. Error bars, SD. *(A)* Welch’s t-test, CBS767 T(2.112)=5.706, P=0.0259; CDA1 T(2.794)=1.412, P=0.2590. *(C)* Multiple unpaired t-tests, CBS767: t(6)=3.949, P=0.008. (D) Multiple unpaired t-tests. CBS767: t(6)=4.976, P=0.003.

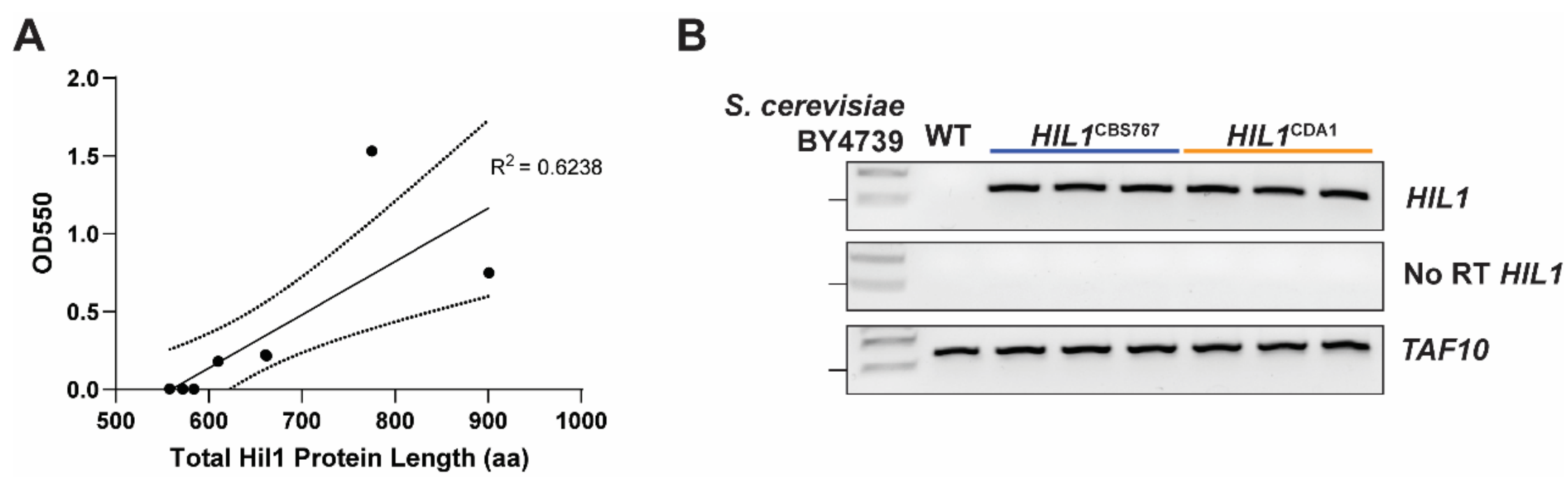

Figure S6. *Correlation of Hil1 length with biofilm formation and validation of heterologous HIL1 allele expression. (A)* Simple linear regression of total Hil1 protein length plotted against quantitated biofilm formation via crystal violet staining for *D. hansenii* strains. Best fit line is solid; 95% confidence intervals were plotted as dotted lines. R^2 goodness of fit = 0.6238. N=11 strains; CCFD4 was excluded due to lack of predicted Hil1 protein. *(B)* SYBR safe stained agarose gel of RT-PCR products. Each lane represents an independent culture of *S. cerevisiae* cells with or without heterologous expression of indicated *D. hansenii HIL1* alleles. *TAF10* is used as a reference transcript. RNA was isolated from mid log cells following five hours of galactose induction. Marker highlights 100bp ladder.

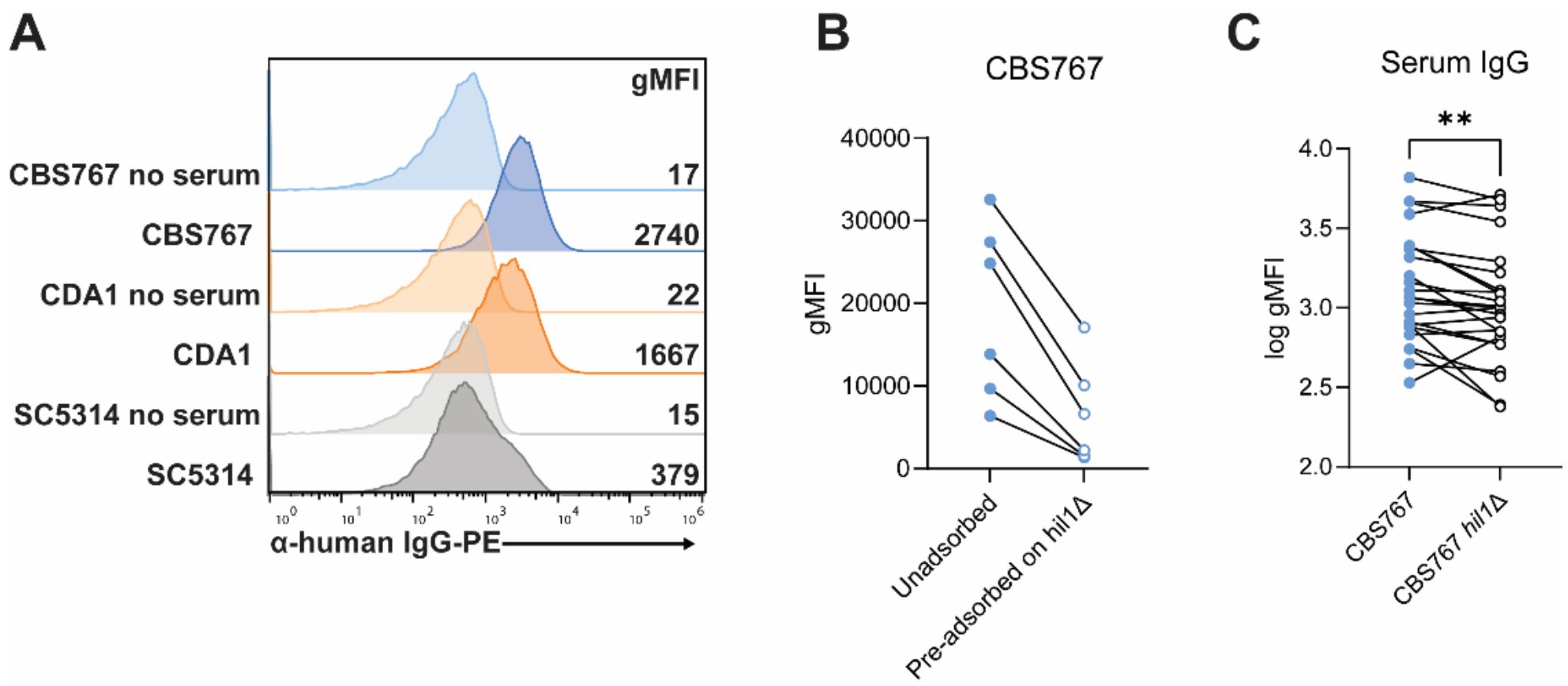

Figure S7. *Validation of CD patient serum IgG binding assay. (A)* Representative histogram from one CD patient. No serum controls were stained with α-human IgG PE secondary antibody only. *(B)* IgG deposition on CBS767 of unadsorbed serum or serum pre-adsorbed with $1x10^8$ CBS767 *hil1*Δ cells to validate adsorption procedure used in Fig. 5B. *(C)* Human serum IgG deposition on yeast cell wall surface of CBS767 or CBS767 *hil1*Δ strains was quantified using flow cytometry N=24 CD patients. For CBS767 *hil1*Δ, each point represents one patient tested on two clones of CBS767*hil1*Δ averaged together. *Statistical Analysis*. ** denotes P<0.01. *(C)* Paired T test, T(23)=3.037, P=0.0059.

Table S1. Mapped coordinates of t-DNA insertion sites in AtMT insertional mutants. Mapping was not successful for all mutants used in the study.

Mutant	Phenotype	Pooled Sequencing Run	Chromosome	Genomic Coordinates	Genetic Context
**B1**	Sm	KN001	G	NC_006049.2: 2004196	Promoter DEHA2G24794g
**C1**	Sm	KN001	G	NC_006049.2: 2,033,147	Coding Sequence of DEHA2g25014g
**E1**	Sm	KN002	C	NC_006045.2: 313,142	Promoter of DEHA2C03388g
**F1 & G1**	Sm	KN002	G	NC_006049.2: 2,022,184	Promoter of DEHA2G24948g
**B2**	Sm	KN003	B	NC_006044.2: 625,748	Promoter of DEHA2B07920g
**C2**	Sm	KN003	G	NC_006049.2: 2,000,803	Coding Sequence of DEHA2G24728g
**E2**	Sm	KN004	G	NC_006049.2: 2,008,028	Promoter of DEHA2g24816 & DEHA2g24838g
**A2**	Wr	KN004	E	NC_006047.2: 970,031	Promoter of DEHA2E12342g

Table S2. List of *Debaryomyces hansenii* strains used in this study.

Strain Identifier	Isolate Type	Pellicle Formation	Colony Morphology	Origin	Isolated From	Source
**CBS767**	Reference Strain, Food	Yes	Wrinkled	Denmark	Sherry	ATCC 36239
**CCFD1**	Food	No	Smooth	Ohio, USA	Cheese	This study
**CCFD2**	Food	Yes	Wrinkled	Vermont, USA	Cheese	This study
**CCFD3**	Food	Yes	Smooth	Wisconsin, USA	Cheese	This study
**CCFD4**	Food	No	Smooth	Michigan, USA	Cheese	This study
**DH-60**	Food	No	Smooth	Wisconsin, USA	Cheese	Banjara et al., 2016
**DH-76**	Food	No	Smooth	Wisconsin, USA	Cheese	Banjara et al., 2016
**DH-242**	Food	Yes	Smooth	Italy	Cheese	Banjara et al., 2016
**DH-246**	Food	Yes	Smooth	Wisconsin, USA	Cheese	Banjara et al., 2016
**CDA1**	Crohn Disease	No	Smooth	Missouri, USA	Patient 1, ileum	Jain et al., 2021
**CDA2**	Crohn Disease	No	Smooth	Missouri, USA	Patient 2, ileum	Jain et al., 2021
**CDE1**	Crohn Disease	No	Smooth	Missouri, USA	Patient 3, ileum	Jain et al., 2021

Table S3. List of oligonucleotides used in this study.

Oligo Name	Oligo Sequence 5'->3'	Primer Pair	Purpose
KN129	**CCACATAAGCTTTCTTACATTTTAC**	KN129+KN133	Amplify left flank (Hil1 terminator)
KN133	**GGCACCTTGTGGTTATGTGATCT**ACCCAACCTTCTTAGTTT	KN129+KN133	Amplify left flank (Hil1 terminator) and overhand to pKN403
KN134	GTTCGGATATATATAAGACT**TGTAATGGAAAAGTTTTCTAATCTTG**	KN143+KN132	Amplify right flank (Hil1 promoter) and overhang to pKN403
KN132	**TTGCGCGTGTTTGATTGTTC**	KN143+KN132	Amplify right flank (Hil1 promoter)
KN135	AACTAAGAAGGTTGGGTAGA**TCACATAACCACAAGGTGCC**	KN135+KN136	Amplify SAT1 resistance cassette and overhang to right flank
KN136	TAGAAAACTTTTCCATTACA**AGTCTTATATATATCCGAACTTGGT**	KN135+KN136	Amplify SAT1 resistance cassette and overhang to left flank
KN137	**ATCCGCCCATGAGTCGATTG**	KN137+KN138	Nested primer to amplify final HDR Cassette
KN138	**AGAGTACATTTACACTCCGGCA**	KN137+KN138	Nested primer to amplify final HDR Cassette
KN100	CATGTTAAAGCGAGGACAATGAAC	KN100+KN101	Amplify deha2g24970g (HIL1) in Figure 4
KN101	GCGCAAACGATTACTGAAAACACAG	KN100+KN101	Amplify deha2g24970g (HIL1) in Figure 4
KN178	GGGGATGAAGCCCAATCCAA	KN178+KN179	Amplify ACT1 (DEHA2D05412g) located within CDS
KN179	GGGGCTTCGGTCAACAAAAC	KN178_KN179	Amplify ACT1 (DEHA2D05412g) located within CDS
KN176	GACGGTTGTTTCAACGTCGG	KN176+KN177	Amplify Hil2 (DEHA2B01452g) located within CDS
KN177	ACCAAAACCGGCCACCTTTA	KN176+KN177	Amplify Hil2 (DEHA2B01452g) located within CDS
KN170	ACAAGATGGTGTCGCATCAGT	KN170+KN171	Amplify HIL3 (DEHA2D00132g) located within CDS
KN171	TGCTTCAAGTACGGACCCAC	KN170+KN171	Amplify HIL3 (DEHA2D00132g) located within CDS
KN168	GTGGGCTCAGGTGGACTTTT	KN168+KN169	Amplify HIL1 (DEHA2g24970g) located within CDS
KN169	GAAGCAATTGTGCATTGGCG	KN168+KN169	Amplify HIL1 (DEHA2g24970g) located within CDS
KN180	GGCATCGTTAGCATTCACCC	KN180+KN181	Genotype crispr mutants; Amplify from genome across HIL1 HDR cassette
KN181	AGGTTTCGTGTTGATGCCCA	KN180+KN181	Genotype crispr mutants; Amplify from genome across HIL1 HDR cassette

KN182	TCGGTGATCCCTGAGCAGGTGG	KN182+KN180	Genotype crispr mutants; confirm SAT1 replacement of HIL1 via HDR, span SAT1 to genome
KN188	tgttatccctagcggatctgacggattagaagccgccg	KN188+KN189	Gibson assembly of *HIL1* heterologous expression cassette
KN189	gggtttttctccttgacgttaaag	KN188+KN189	Gibson assembly of *HIL1* heterologous expression cassette
KN190	acgtcaaggagaaaaaccccctgcagctcgagggatcc	KN190+KN191	Gibson assembly of *HIL1* heterologous expression cassette
KN191	ttacctaggagatctgtctcatgttaaag	KN190+KN191	Gibson assembly of *HIL1* heterologous expression cassette
KN192	gagacagatctcctaggtaataaggcgcgccacttctaaataag	KN192+KN193	Gibson assembly of *HIL1* heterologous expression cassette
KN193	cagatccgctagggataacag	KN192+KN193	Gibson assembly of *HIL1* heterologous expression cassette
KN216	ATATTCCAGGATCAGGTCTTCCGTAGC	KN216+KN217	Amplify *TAF10* from *S. cerevisiae* as reference gene for RT-PCR (Teste et al., 2009)
KN217	GTAGTCTTCTCATTCTGTTGATGTTGTTGTTG	KN216+KN217	Amplify *TAF10* from *S. cerevisiae* as reference gene for RT-PCR (Teste et al., 2009)
DhPadF	GCGACTATGAACAGGTTTCC AACGA	DhPadR + DhPadF	*D. hansenii* discriminating primer (Wrent et al., 2015)
DhPadR	CCTTCAATGTAACATCAGCGGCCC	DhPadR + DhPadF	*D. hansenii* discriminating primer (Wrent et al., 2015)
ITS1F	CTT GGT CAT TTA GAG GAA GTA A	ITS1F +ITS4	Amplify fungal ITS1-5.8srRNA-1TS2 locus (Raja et al., 2017)
ITS4	TCC TCC GCT TAT TGA TAT GC	ITS1F +ITS4	Amplify fungal ITS1-5.8srRNA-1TS2 locus (Raja et al., 2017)
DhTEF11	CTGAGAGGGCCCAGTCTTATATATATCCGAACTTGG	DhTEF11+DhTEF12	DH TEF1 promoter, clai cutsite, published in (Defosse et al., 2018)
DhTEF12	CCTGCAGGATCGATTTTGATTAATTAATATATAAAATATATG	DhTEF11+DhTEF12	DH TEF1 promotoer, apai cutsite, published in (Defosse et al., 2018)
HIL1 gRNA 1	TTGTAACATAGAACCCACCA	NA	CRISPR HIL1 PAM CCG, (+) strand
HIL1 gRNA 2	GGGGGCAATCTTGATAACCG	NA	CRISPR HIL1, PAM TGGTGG, (-) strand
